# Implementation of the Freely Jointed Chain Model to Assess Kinetics and Thermodynamics of Thermosensitive Coil–Globule Transition by Markov States

**DOI:** 10.1021/acs.jpcb.1c01946

**Published:** 2021-05-04

**Authors:** Patrick K. Quoika, Monica L. Fernández-Quintero, Maren Podewitz, Florian Hofer, Klaus R. Liedl

**Affiliations:** Institute of General, Inorganic and Theoretical Chemistry, and Centre of Molecular Biosciences University of Innsbruck, A-6020 Innsbruck, Austria

## Abstract

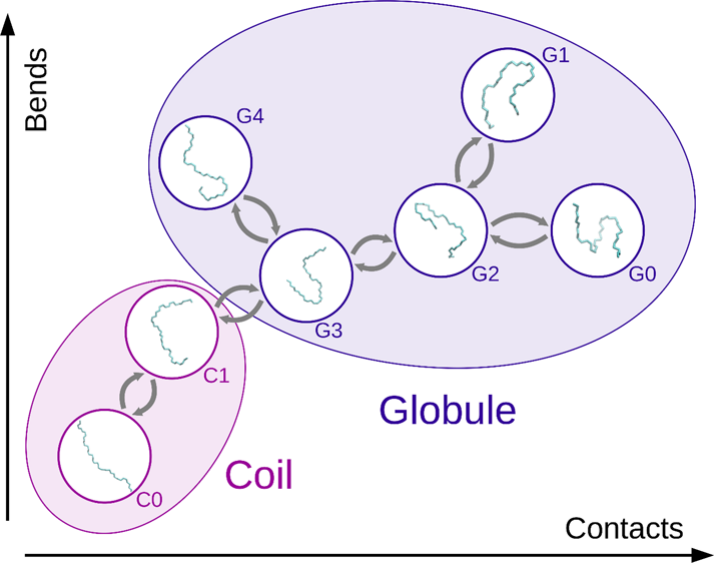

We revived and implemented a method developed by Kuhn in 1934, originally only published in German, that is, the so-called “freely jointed chain” model. This approach turned out to be surprisingly useful for analyzing state-of-the-art computer simulations of the thermosensitive coil–globule transition of *N*-Isopropylacrylamide 20-mer. Our atomistic computer simulations are orders of magnitude longer than those of previous studies and lead to a reliable description of thermodynamics and kinetics at many different temperatures. The freely jointed chain model provides a coordinate system, which allows us to construct a Markov state model of the conformational transitions. Furthermore, this guarantees a reliable reconstruction of the kinetics in back-and-forth directions. In addition, we obtain a description of the high diversity and variability of both conformational states. Thus, we gain a detailed understanding of the coil–globule transition. Surprisingly, conformational entropy turns out to play only a minor role in the thermodynamic balance of the process. Moreover, we show that the radius of gyration is an unexpectedly unsuitable coordinate to comprehend the transition kinetics because it does not capture the high conformational diversity within the different states. Consequently, the approach presented here allows for an exhaustive description and resolution of the conformational ensembles of arbitrary linear polymer chains.

## Introduction

Thermosensitive polymers have been of major interest in many fields of research.^1^ Besides various medical applications, for example, drug carriers and synthetic tissues, they have also been proven to be applicable in gel actuators and oil refineries.^2−5^ Interestingly, thermosensitive polymers undergo a phase transition with a lower critical solution temperature (LCST).^6,7^ Indeed, this phase transition is connected to a conformational change of the polymer chains, that is, the coil–globule transition (CGT).^8,9^ Possibly, the most prominent example of such a polymer is *N*-Isopropylacrylamide (NIPAAM).^10,11^ A schematic visualization of the CGT and the chemical formula of the NIPAAM monomer are given in the supporting information. Since its discovery, the thermosensitive CGT has been the research object for a large community of scientists, both experimentally and computationally. Still, the origins of this transitions are not fully understood yet.

Even though the CGT is often compared to protein folding, some crucial differences may be identified. Certainly, both these processes describe the conformational change from an extended to a collapsed state. However, the CGT lacks a precise conformational definition of these states. In contrast to protein folding, there is nothing like a native fold, which may be assumed to be a global energetic minimum and therefore serve as a convenient reference state. In experiments, both conformational states of the polymer, that is, extended and collapsed, are merely distinguished by their size distribution. Therefore, both states — which are conventionally referred to as coil and globule — exhibit a large variety of conformations.

Molecular dynamics (MD) simulations have been established as state-of-the-art to capture conformational ensembles of macromolecules of diverse nature on an atomistic level.^12−14^ However, in computational studies, the characterization of the CGT of thermosensitive polymers has been shown to lead to some technical challenges: because the conformational space of both states — coil and globule — is large, extensive sampling is crucial. Besides, the time scale of the conformational transition is slow, and in addition, simulations over a wide range of temperatures are necessary. Conclusively, similar as for protein folding, substantial computational effort needs to be invested to obtain reliable reconstructions of the thermodynamics and kinetics of the CGT*.*

Furthermore, an optimal reaction coordinate for an accurate description of the process — which captures all relevant degrees of freedom^15,16^ — is lacking.^17^ While the two conformational states, that is, coil and globule, are conveniently defined by their size distributions in experiments, such a simple description is not fully sufficient *in silico*. Commonly, the states are identified by means of the radius of gyration *(R*_g_) because this observable is accessible in experiments and intuitively interpretable. Nevertheless, it is an insufficient quantity to describe the transition in terms of free energy: looking at *R*_g_ alone, states which are conformationally very different may be projected onto one another. Therefore, energetically favorable conformations may be consolidated with relatively unfavorable conformations.^17^ For example, structures on the transition path to a certain globular conformation — which are therefore short-lived — may exhibit the same *R*_g_ as a totally different conformation. The latter may in comparison be more stable. Indeed, it may even be a globular conformation by itself. Projecting these structures onto the same value of *R*_g_ may result in a smeared-out free energy curve. Certainly, conformations of equal *R*_g_ need to be neither thermodynamically equivalent nor similar in terms of dynamics. On the contrary, they may be rather different: a certain class of globules may exhibit more internal interactions and therefore less interactions with water in comparison to another class of conformations with the same *R_g_*.^18^ Conclusively, the energy and also the entropy difference associated with the conformational change may differ between these classes. Analogously, semi-stable misfolded proteins are usually assumed to be different from their native structure with respect to thermodynamics, despite exhibiting the same *R*_g_*.*(16,19−21)

In prior publications, we found that thermodynamic quantities, such as the enthalpy, may vary significantly between repeated simulations of the CGT.^17,18^ Conclusively, we presumed the conformational diversity in both states to be of particular importance. Besides, we were convinced that the thermosensitive character of the CGT originates from an entropic effect.^18,22−24^ Specifically, we assumed that a large variety of globular structures exist, whose properties show significant deviations in terms of thermodynamics and kinetics. However, as of now, distinguishing between conformational substates has been challenging due to the lack of suitable descriptors. On top of that, detailed information about polymer conformations is challenging to obtain experimentally. Nevertheless, experimental evidence for conformational substates has been found.^25,26^ Indeed, indications for multiple metastable states in both ensembles have been found in coarse-grained simulations.^27^ Accordingly, to account for the diversity of conformational substates, these need to be identified unambiguously. Thus, it was our goal to find a small number of comprehensible descriptors to this end.

We performed a detailed analysis of the thermodynamics and the kinetics of the CGT at different temperatures. Due to the long transition time scales and the necessity for an extensive conformational ensemble, we performed very long simulations. In fact, we invested multiple magnitudes of the computation time of previous studies to allow for reasonable estimates of the thermodynamics and the kinetics of the process at different temperatures. Furthermore, to facilitate exhaustive sampling of the conformational dynamics of the polymer, we chose to simulate the NIPAAM 20-mer because a polymer chain of this length already shows the CGT, while exhibiting a tremendously smaller conformational space than longer polymer chains.^28,29^ Furthermore, according to previous studies of the dependence of the CGT on the polymer length, the 20-mer is suited to draw general conclusions about the process.^30^ Conclusively, the 20-mer represents the ideal length for our study (for a more detailed discussion, see supporting information).

Moreover, we used a newly developed tool to rapidly compute the conformational entropy of the polymer chain from the angular distribution of the backbone dihedrals,^31^ which is published elsewhere.^32^ Further, to verify the hypothesis that the CGT comprises transitions between distinct substates*,* we used polymer-specific descriptors to resolve the conformational diversity. To this end, we employed the freely jointed chain model.^33,34^

## Computational Methods

### Simulation Setup

As starting structures for the MD simulations, we prepared extended conformations of syntactic 20-mers of NIPAAM. To this end, we used the Maestro software package.^35^ We solvated these structures in cubic boxes of a side length of 7 nm with extended single point charge water model (SPC/E).^36,37^ Prior to the MD simulations, we minimized the energy of the initial configurations with the steepest descent method. Furthermore, before the production runs, we equilibrated the system in short simulations with constant volume. Except for the preparation of the initial polymer configuration, we used the GROMACS MD-Simulation software package throughout this process.^38^ For all simulations, we used the OPLS2005 force field,^39,40^ which has been established for simulations of NIPAAM in past publications.^17,18,41−45^ In our production runs, we applied the Parrinello−Rahman barostat,^46,47^ with a reference pressure of 1 bar and the velocity-rescaling thermostat^48^ at respective simulation temperatures between 250 and 360 K. We used the LINCS algorithm to constrain the bonds involving hydrogen atoms and used a timestep of 2 fs for our MD integration.^49,50^ Throughout all simulations, we applied periodic boundary conditions. Furthermore, we used a cutoff of 8.85 Å for the evaluation of long-range interactions and applied the particle mesh Ewald method to this end.^51^

At all temperatures, we performed multiple simulations with a length of 5 μs each. At temperatures close to the expected CGT temperature (T*), that is, between 260 and 310 K, we simulated 12 replicas each. To save computational effort, we used fewer replicas at temperatures far from the expected T* (below 260 K and above 310 K). Accordingly, these temperatures were not included in the analyses. The number of replicas and the total simulation length at different temperatures is visualized in the supporting information.

### Thermodynamic and Kinetic Analysis

#### Free Energy and Equilibrium Constants

Generally, we obtained a projection of the free energy of the CGT from the distribution of the radius of gyration (*R*_g_) in our MD simulations, employing Boltzmann’s distribution.^52,53^ Furthermore, we separated the conformational ensemble, which we obtain from all replicas at a specific temperature into the coil and globule subsensembles, respectively. Consequently, we calculate the equilibrium constant, *K*_eq_, from the ratio of probabilities to find the polymer to be either in coil or globule conformation. Thus, we calculate the free energy of the CGT (Δ*G*) as follows:^52,53^
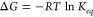
1where *R* is the ideal gas constant and *T* is the temperature of the respective simulation. Following this approach, we obtain the free energy, Δ*G*, of the transition at different temperatures, that is, the free energy difference of the coil and globule states at the respective temperature. Therefore, we separated the subsensembles by means of a two-state Markov state model (MSM, see below).

To be able to separate energetic contributions to Δ*G*, we evaluate the temperature dependence of *K*_eq_ according to the van’t Hoff equation,^54^
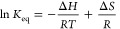
2where R denotes the ideal gas constant and T is the temperature. Furthermore, we assume Δ*H* and Δ*S* — the differences of coil and globule in enthalpy and entropy, respectively — to be independent of the temperature. This appeared to be a valid approximation within a certain variance. In fact, we observed the temperature independence of the enthalpy of the CGT in previous studies.^18^ Besides, this assumption may be verified by assessing the linearity of the van’t Hoff plot,^55^ which we depict in the supporting information.

#### Polymer Entropy

We assume the torsional degrees of freedom of the polymer backbone to dominate the entropy of the polymer. Hence, we neglected other eventual internal degrees of freedom, such as bond angles, but also dihedrals of the side chains. Therefore, we assessed the distribution of all torsional degrees of freedom of the polymer backbone at different temperatures. Furthermore, we separated coil and globule conformations and processed the ensembles separately. Thus, we estimated the torsional entropy by integrating the distribution of a specific torsional degree of freedom in the respective state.^31,56^ Therefore, we estimated the probability density with a kernel density estimation. We used a newly developed hybrid Python/C++ implementation for that, which is published elsewhere.^32^ Conclusively, we calculated the difference in dihedral entropy for all backbone dihedrals independently and summed them up to obtain the CGT entropy of the polymer, Δ*S*_Pol_.

#### Transition Rates

In order to evaluate the kinetics of the CGT, we divided the simulation ensembles at different temperatures in two conformational states. To this end, we employed hidden Markov state models (see below). Furthermore, we calculated the transition rate from the obtained mean first passage times of these stochastic models. We expected the temperature dependence of the transition rate of the CGT to follow to the Eyring equation^57^
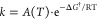
3where *R* is the ideal gas constant, Δ*G*^†^is the free energy difference to the transition state, *T* is the temperature, and *A(T)* is a temperature-dependent pre-exponential factor. We modeled the temperature dependence of *A*, which is often referred to as the frequency factor, linearly, *A*(*T*) = *c* · *T*. Here, the constant *c* estimates the mean frequency with which the system approaches the transition state barrier.

### Conformational Description

In order to identify structures which are similar in *R*_g_, but very different in conformation, we developed specific structural descriptors for linear polymers. Thus, we modeled the polymer as a freely jointed chain.^33,34,58,59^ Usually, freely jointed chains are modeled with a segment length of twice the persistence length, that is, the Kuhn length.^33^ Nevertheless, we decided to use comparably short segments to guarantee the capturing of all conformational information. Accordingly, we modeled segments of the length of the persistence length.^58,60^ In accordance with prior publication, we applied a segment length of 3 monomer units.^18^

Additionally, we calculated angles between these segments, as well as centers of masses of these segments. We used the latter for evaluating the distance between these segments. Therewith, we were able to quantify eventual contacts between these segments for a given conformation. The calculation of the two resulting descriptors, that is, the sum of angles between the segments (Ω) and the number of contacts between the segments (ν), is elaborated on in the supporting information. The post-processing of the simulations has been implemented in Python, and it strongly employs the modules: NumPy,^61^ SciPy,^62^ and MDAnalysis.^63^

### Markov State Models

Generally, we built MSMs with two different purposes: first, to generally distinguish coil and globule conformations at different temperatures; second, to identify subensembles within these two states. Depending on the purpose, the level of detail of these models is different. We elaborate on both procedures below. Generally, we extensively used the PyEMMA python package for these analyses.^64^

#### Macrostate Assignments

With a previously published method, we were able to classify polymer conformations based on *R*_g_ and σ (solvent accessible surface area). Thus, we were able to identify conformations which are either clearly globules or clearly coils.^17,18^ However, this method was not able to distinctly classify a certain small proportion of conformations. Here, we used hidden MSMs (hMSMs) to resolve the classification of these conformations based on the dynamics of the system. To this end, we grouped the nondistinctly classified structures in small clusters and employed the MSM to assign these structures to one or the other macrostate based on the conformational transitions in the simulation. This procedure is described in more detail in the supporting information.

#### Conformational Substates

##### Preprocessing

In order to build consistent MSMs at different temperatures, we performed a global *k*-means clustering in four dimensions. Therefore, we processed conformations from simulations at all temperatures. To identify conformational substates, we described the polymer conformations by means of *R*_g_, σ, Ω, and ν, that is, the radius of gyration, the solvent-accessible surface area, and the sum of the angles between the polymer segments and the number of contacts between these segments. Furthermore, to facilitate a good localization of our MSM in this four-dimensional space, we used 250 clusters, which we obtained with the *k*-means algorithm implemented in scikit-learn.^65^ To prevent the clustering from being dominated by the eventually larger scale of a certain dimension in this space, we applied a standard scaling on all coordinate dimensions. Prior to building of the MSM, we assigned every conformation in our trajectories to one of these clusters.

##### MSM Building

In order to resolve substates within the two macrostates, that is, coil and globule, we made use of the MSM method. Therefore, we built independent hMSMs for all temperatures. Because timescales are generally temperature-dependent, we decided to adapt the lag time for the model building at different temperatures accordingly. The used lag times at the respective temperatures are shown in the supporting information.

## Results

First, we show the thermodynamic and kinetic characterization of the system. In this context, we only considered the two metastates, that is, coil and globule. Subsequently, we show the results of our polymer-specific analysis of the conformational space at different temperatures. Therewith, we show the existence of distinct conformational substates at different temperatures.

### Thermodynamic and Kinetic Analysis

Besides the projection of the free energy on *R*_g_, we analyzed the temperature dependence of the equilibrium constant of the CGT. Furthermore, we performed an analogous analysis of the kinetics of the forward and back transition of the conformational transition. Lastly, we show the conformational entropy of the polymer at different temperatures within the ensembles of the two conformational states, that is, coil and globule.

**Figure 1:**
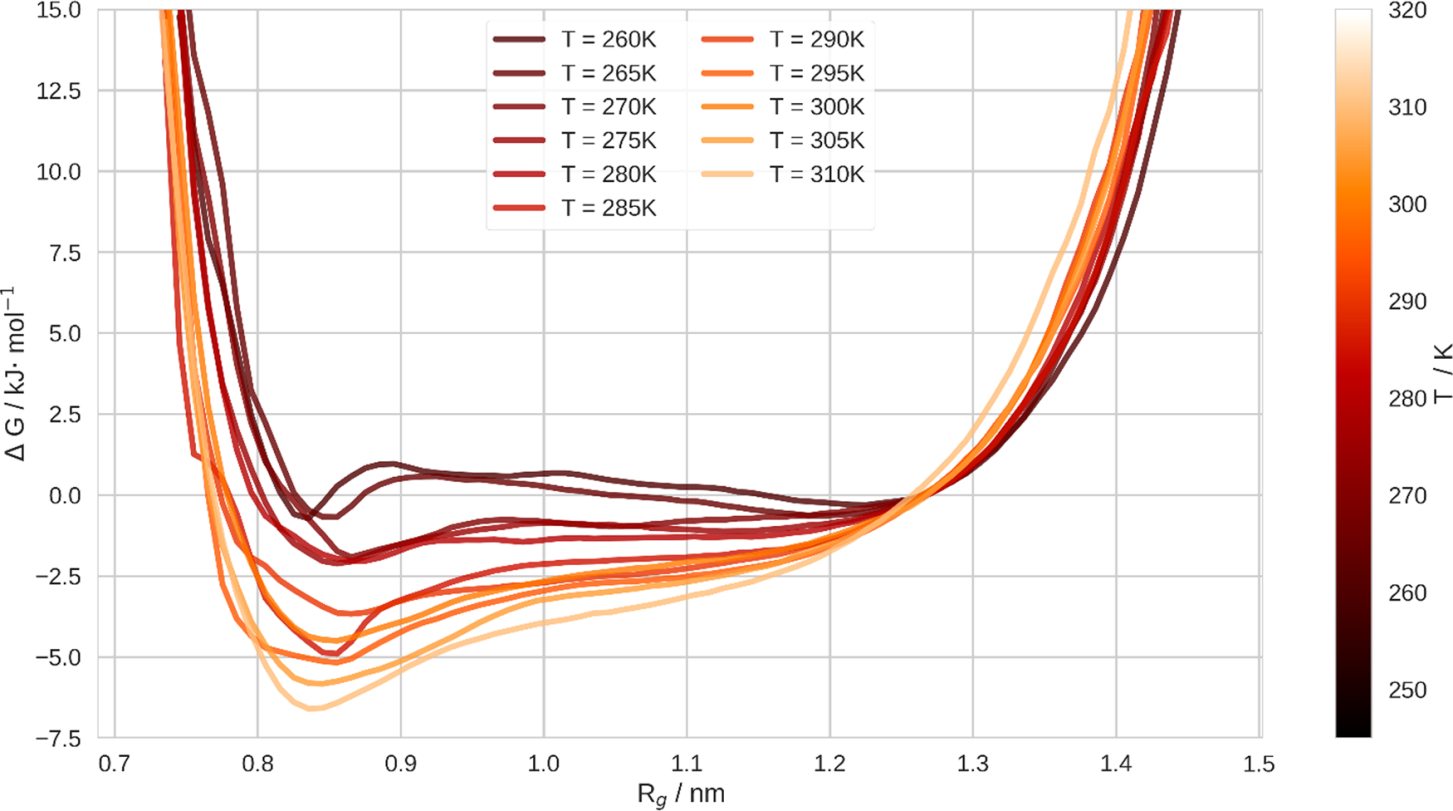
Projection of the free energy of the CGT at different temperatures on the radius of gyration (*R*_g_). The curves are colored according to the temperature. The curves have been aligned by performing a parabolic fit of the flank of the curves at large *R*_g_.

We projected the free energy of the CGT at different temperatures on the *R*_g_ of the polymer in the simulations, as shown in Figure 1. Therefore, we included all 12 replicas of each 5 μs at the respective temperature. Ergo, we evaluated 60 μs at all these temperatures. To facilitate the comparison of the progression of these curves, we performed a parabolic fit of the right flank of these curves and shifted them accordingly. Furthermore, we color-coded the curves according to the temperature. Consistently, we observe that the higher the temperature, the lower the free energy in the local minimum at low *R*_g_ — which corresponds to the conformational state of the globule. Furthermore, we note that the barrier between the two local minima is not very pronounced in this projection of the free energy. Apparently, it even vanishes completely at high temperatures. As a result, the generally broad minimum of the coil state appears to be rather shallow in this projection.

**Figure 2:**
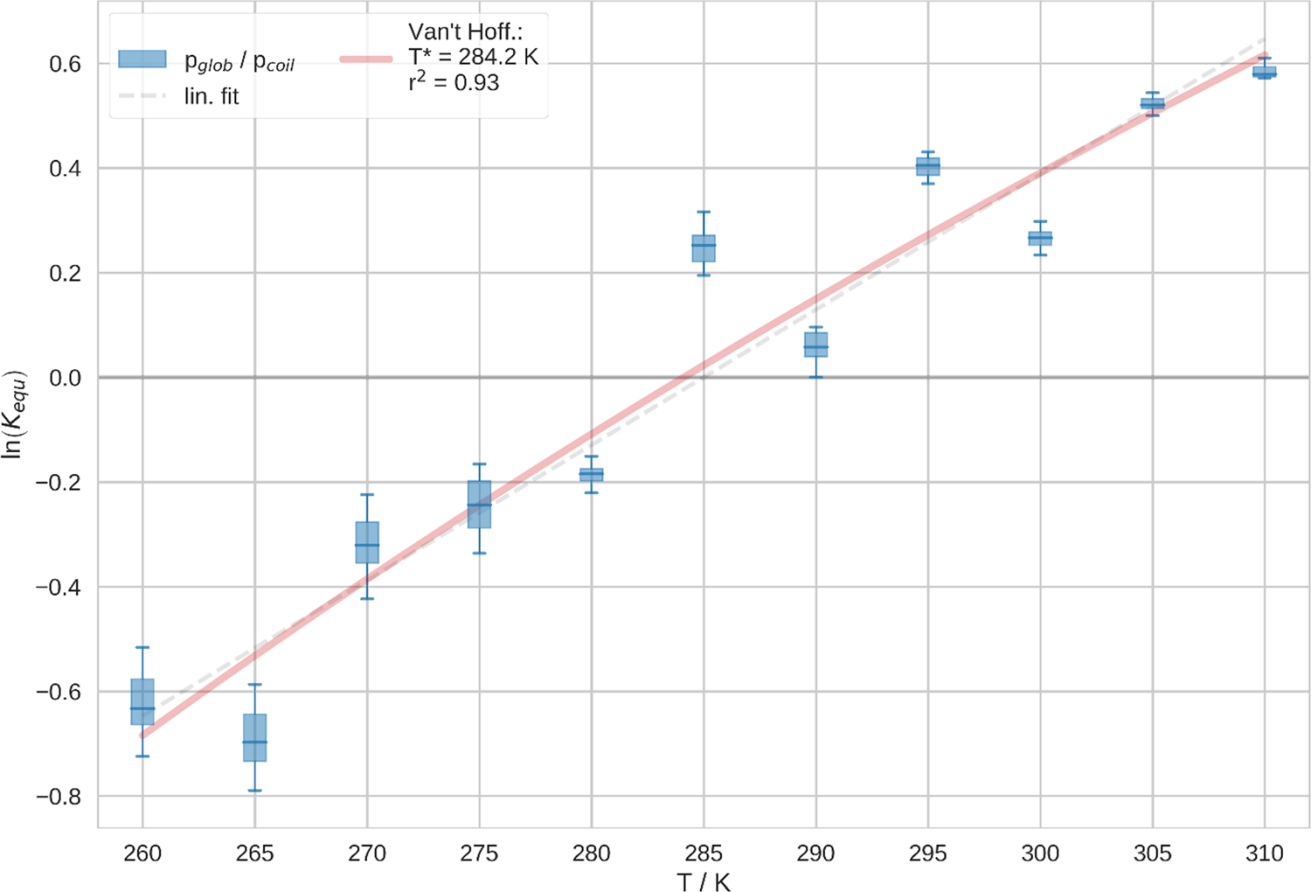
Equilibrium constants of the CGT at different temperatures, *K*_equ_ = *p*_glob_/*p*_coil_, obtained from two-state hMSMs at the respective temperature. Hyperbolic fit according to the van’t Hoff equation. We show uncertainties from LOO as a box and whiskers plot: the boxes depict the range of the data within the second and the third quartile. Furthermore, the whiskers depict min and max values of the LOO.

Furthermore, we analyzed the equilibrium constants of the CGT at different temperatures, as shown in Figure 2. To this end, we calculated the ratio of the number of conformations classified as globules over the number of those classified as coil in the simulations at the respective temperatures. These structures have been assigned in accordance with the two-state MSMs as explained above. We estimated the uncertainty of these equilibrium constants by Leave-One-Out (LOO) cross-validation. Thus, we iteratively excluded single replicas from the analysis and compared the change of the obtained results with the remaining replicas. We note that, as a trend, the uncertainty is smaller at high temperatures. Furthermore, we performed a hyperbolic fit of these values according to the van’t Hoff equation. This fit exhibits good agreement with the data, that is, *r*^2^ = 0.93.

**Figure 3:**
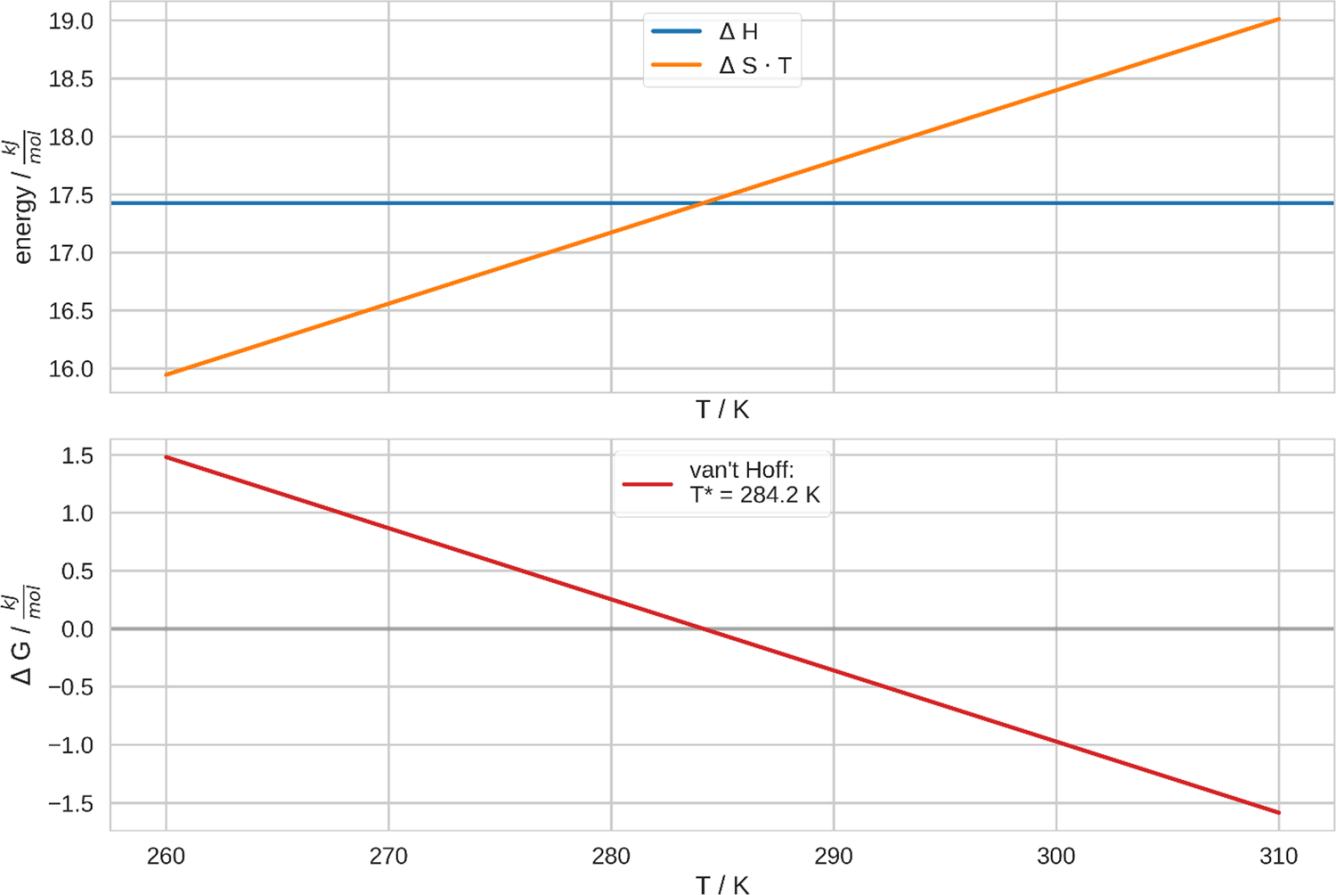
Contributions to the free energy of the CGT. Here, we show the results from the van’t Hoff fit in Figure 2. In the upper panel, we show the enthalpy and the entropy of the CGT at different temperatures. Therefore, we plot the entropy times the temperature. In the lower panel, we show the respective free energy at different temperatures. Accordingly, the CGT transition temperature has been determined as T* = 284.2 K.

Moreover, in Figure 3, we show the free energy of the CGT, Δ*G*, which may straightforwardly be calculated from the equilibrium constants, according to eq 1. We show the dependence of this quantity on the length of the simulation time per replica for different temperatures in the supporting information. Generally, the convergence of Δ*G* strongly depends on the simulation temperature. We found that shorter simulation times may potentially suffice at high temperatures, whereas at low temperatures, such long simulations are necessary to obtain an accurate estimate of Δ*G*.

We evaluated the enthalpy and entropy according to the van’t Hoff fit, which we conducted as shown in Figure 2. Here, we assumed the temperature dependence of the differences in enthalpy and entropy of coil and globule to be negligible in this range of temperatures. Accordingly, Δ*H* is constant in this plot, while Δ*S* · *T* increases linearly with the temperature. Conclusively, Δ*G* decreases linearly. Consequently, it changes sign at the transition temperature, T* = 284.2 K. These assumptions have been validated in the van’t Hoff plot. There, we assessed the linearity of the curve after linearization of the data (see SI). As an additional validation, we also performed an analogous analysis, assuming both Δ*H* and Δ*S* to be explicitly temperature dependent, which led to equivalent and consistent results. We show this alternative evaluation in the supporting information. Because this analysis is based on the equilibrium constants, it considers the free energy of the whole system. Therefore, these values comprise eventual contributions of the solvent to the free energy.

**Figure 4:**
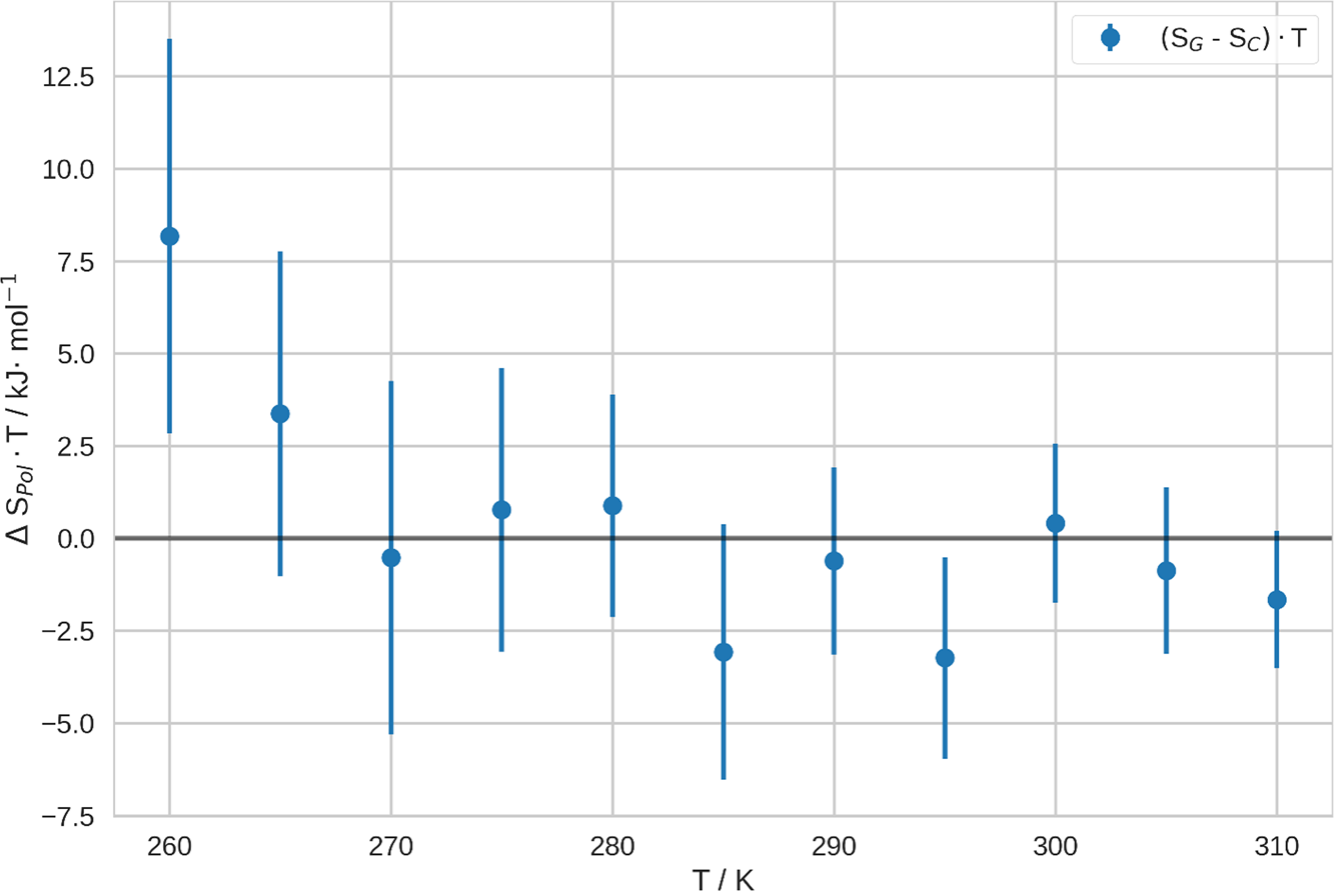
Difference in conformational entropy of the coil and globule ensembles at different temperatures, Δ*S*_Pol_ = *S*_G_ – *S*_C_, multiplied by the respective temperature. These quantities have been obtained from the probabilty densities of the dihedral angles along the backbone of the polymer. We evaluated the uncertainty of the entropy within the respective ensemble by LOO. Consequently, the uncertainty of the difference of these values is the sum of the uncertainties of the single value (Gaussian error propagation).

Additionally, we show the contribution of the conformational entropy of the polymer to the free energy in Figure 4. There, we show Δ*S*_Pol_ · *T*, which we estimated from the torsional degrees of freedom of the polymer backbone. Therefore, we processed the coil and globule ensemble separately. Thus, we calculated the entropies and uncertainties of every torsional degree of freedom separately and summed up these values. As a result, we obtain the conformational entropies in both states, that is, coil and globule. Furthermore, we subtracted these entropies at different temperatures, Δ*S*_Pol_ = *S*_G_ – *S*_C_. Further, we multiplied them with the respective temperature. As mentioned above, we estimated the uncertainty of these entropy values by LOO. Generally, the higher the temperature, the smaller is the uncertainty of Δ*S*_Pol_ · *T*. Moreover, we note that this quantity shows a quasi-asymptotic trend. For a wide range of temperatures, Δ*S*_Pol_ · *T* scatters around ca. −1 kJ/mol. This property is only at low temperatures significantly above zero. Conclusively, this contribution energetically disfavors the CGT at most temperatures, however only with a comparably small contribution.

**Figure 5:**
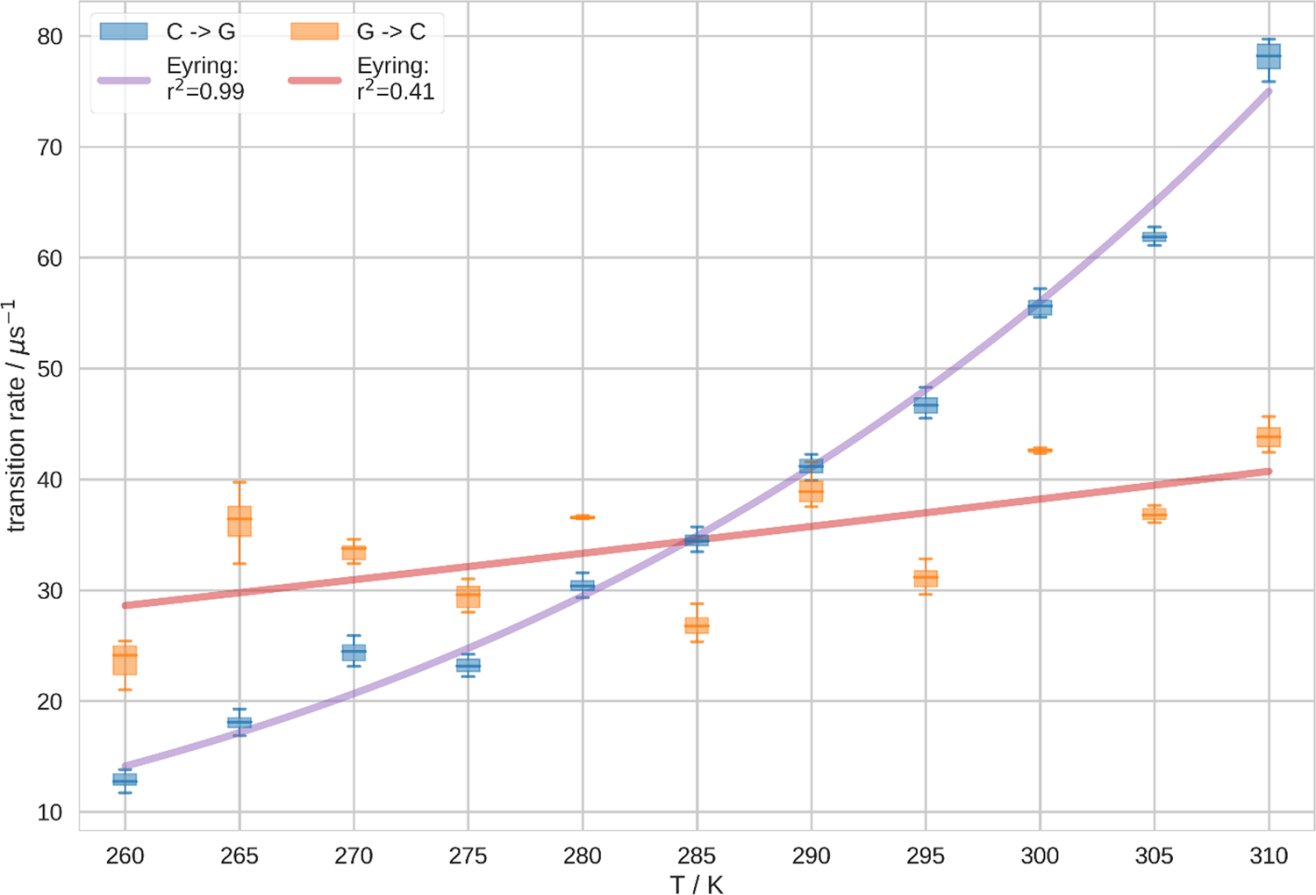
Transition rates for the coil–globule and the globule–coil transition at different temperatures for hMSM. We performed a LOO to quantify the uncertainty of these results. We show these uncertainties in the following way: the boxes depict the range of data of the second and third quartile, and the whiskers represent min and max values. Furthermore, we performed an exponential fit according to the Eyring equation.

In order to evaluate the kinetics of the CGT, we calculated reaction rates from the estimated mean first passage times from the hMSMs at different temperatures. We show the rates of the forward and back transition in Figure 5. We evaluated the uncertainty of these results by LOO. Consequently, we visualized the variance of these results as a box and whiskers plot: In boxes, we show the second and third quartile and the whiskers represent min and max values of the results. In accordance with the Eyring equation, eq. 3, we applied an exponential fit to these curves. We note that while the reaction rates of the CGT agree very well with the expected behavior, that is, *r*^2^ = 0.99, the reaction rates of the back transition exhibit a poor exponential fit, *r*^2^ = 0.41. Further, we notice that the temperature dependence of the latter is generally much weaker. Consequently, the CGT happens at a lower rate than the back transition at temperatures below 285 K. In contrast, at temperatures above, it exhibits a consistently much higher rate. Finally, we notice that the deviation from the fit is generally larger at low temperatures. Furthermore, as a weak tendency, the spread of the estimated reaction rates is larger at low temperatures.

### Resolving Substates by Conformational Descriptors

**Figure 6:**
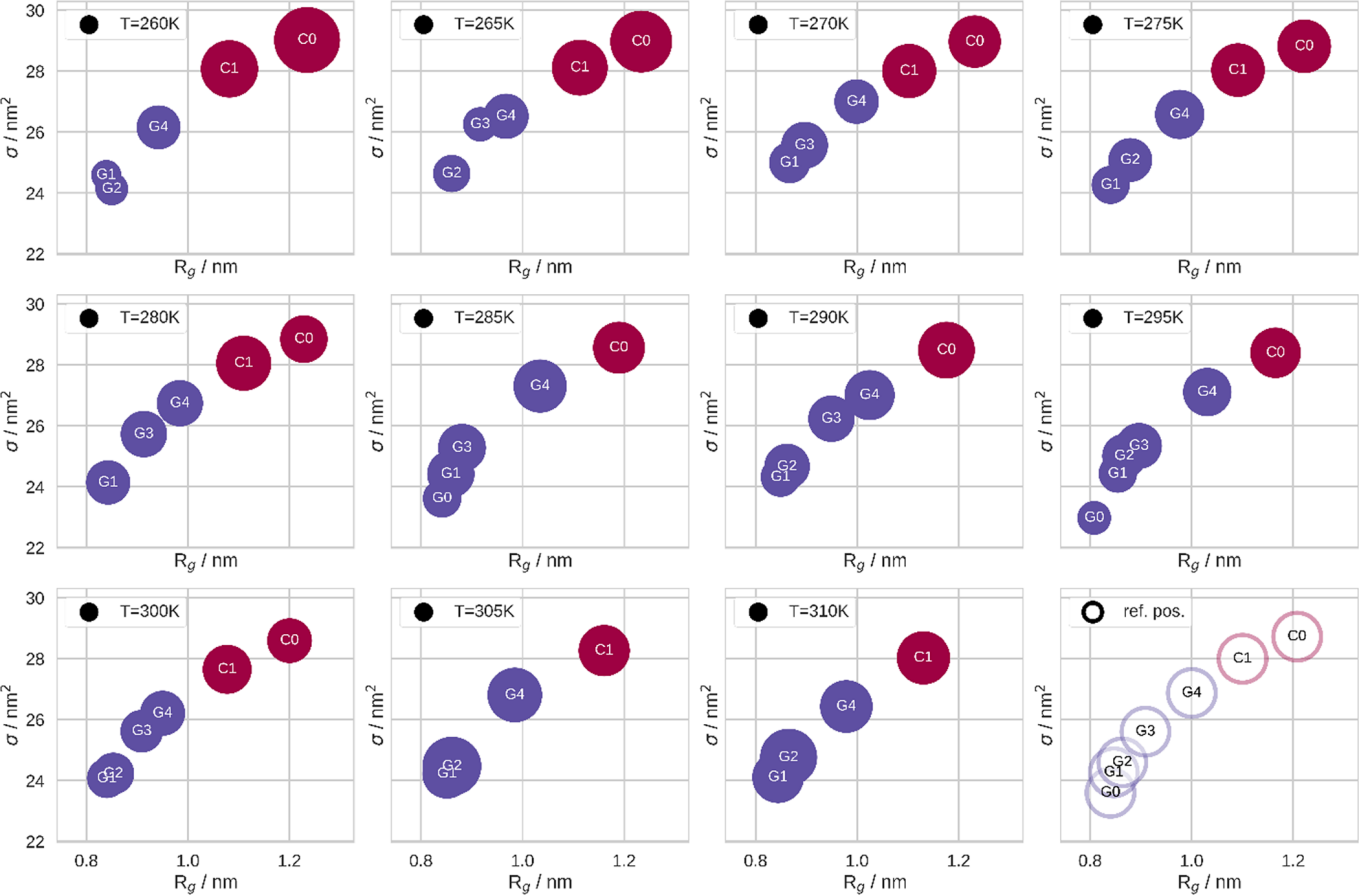
Model of the conformational dynamics of the NIPAAM 20-mer at different temperatures in the *R_g_*-σ plane. States classified as coil are depicted in dark red, and those classified as globule are depicted in purple. The size of the circles corresponds to the occupancy of the state at the respective temperature. In the lower right panel, we show conformational substates of the NIPAAM 20-mer. There, we show the reference positions of the seven conformational states, which we have consistently observed within our simulations at different temperatures.

**Figure 7:**
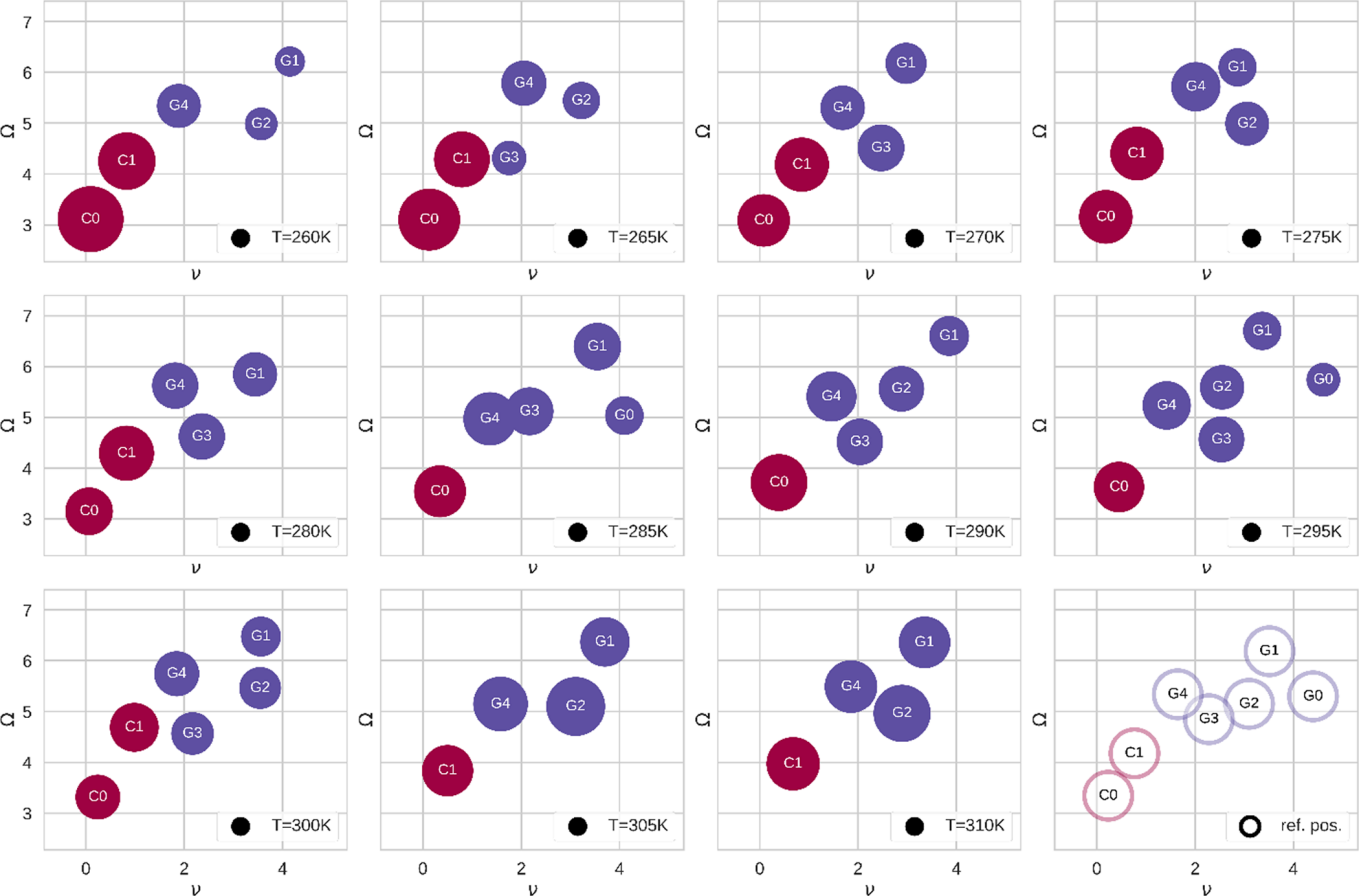
Model of the conformational dynamics of the NIPAAM 20-mer at different temperatures in the ν-Ω plane. States classified as coil are depicted in dark red, and those classified as globule are depicted in purple. The size of the circles corresponds to the occupancy of the state at the respective temperature. In the lower right panel, we show conformational substates of the NIPAAM 20-mer. There, we show the reference positions of the seven conformational states, which we have consistently observed within our simulations at different temperatures.

Below, we show stochastic models of the conformational dynamics of the polymer chain at different temperatures. The conformational substates therein have been determined in a four-dimensional space. Therefore, we depict their position first in the *R*_g_-σ space, as shown in Figure 6, and second in the ν-Ω space, as shown in Figure 7. There, we illustrate states which belong to coil in dark red and states which belong to globule in purple. The occupancy of these substates is encoded in the size of the respective circles.

Generally, we note that the conformational substates consistently reoccur at different temperatures. However, hardly any can be found at all temperatures. In order to compare the models from different temperatures, we defined reference positions for the substates. Therewith, we can provide a consistent and comprehensive identification of the states. Furthermore, we introduced a simple naming scheme for the states: depending on the metastate they belong to, that is, coil or globule, we call them either “*C*” or “G” plus an integer which relates to the *R*_g_ rank of this substate within the metastates. Accordingly, *C0* is the state with the highest *R*_g_, while *G0* is the state with the lowest *R*_g_, which we observed in the simulations. The reference positions are depicted in the lower right corner of Figure 6 and Figure 7. Furthermore, we noticed that these states drift with changing temperatures. Additionally, we note that the states are much better separated in ν-Ω space than in *R*_g_-σ space. In fact, trying to model the conformational substates solely in the latter two coordinates fails. Furthermore, we want to emphasize that looking at the position of the globule states in *R_g_* alone, they are generally very close, even indistinguishable in many cases.

The occupancy of most states exhibits a clear trend with temperature. Generally, we notice a trend of increasing occupancy of the globule states and decreasing occupancy of the coil states, the higher the temperature. Furthermore, we observe, for example, that *G1* consistently reoccurs at all temperatures above 270 K. Moreover, the higher the temperature, the lower is the occupancy of *C0,* until it vanishes at temperatures above 300 K. Interestingly, the only globule which occurs at all simulated temperatures is *G4* — the globule with the highest *R*_g_. In fact, according to the state determination scheme which we used in past publications, *G4* would have been classified as a somewhat intermediate conformation — a class of conformations which are not clearly identified as coil or globule. On the other hand, *G0* is generally rare. Therefore, it presents an exception to the general trend.

## Discussion

Below, we discuss the results of the thermodynamic and kinetic characterization of the simulations first, before we turn toward the identification of the conformational substates at different temperatures. Therefore, we discuss the following aspects separately: first, we address the free energy of the CGT at different temperatures and the connected properties, that is, the entropy and the enthalpy as well as the equilibrium constant, respectively. Second, we elaborate on the polymer entropy, which we resolved separately for both conformational states, that is, coil and globule, at different temperatures. Lastly, the CGT kinetics and its temperature dependence. Afterward, we discuss the conformational substates, which we identified by means of the freely jointed chain model.

### Thermodynamics and Kinetics

Having invested a tremendous amount of computational effort, that is, 60 μs per simulated temperature, we obtained a smooth projection of the free energy of the CGT on *R*_g_ at all temperatures. Generally, these results are consistent with previous publications.^17,18^ As a consequence of our extensive sampling, we report back-and-forth transitions between the two conformational states in all simulation replicas above 280 K. (In fact, also in many simulation replicas below this temperature). These transitions are visible in the time series of *R*_g_ of the polymer, which we show for all replicas at all temperatures in the supporting information. It is noteworthy that the conformational states are generally not separated by a very distinct barrier. This may be explained by the fact that kinetically important degrees of freedom are not considered in this projection.^15^ Accordingly, conformations which lie in one specific local minimum are projected onto other conformations, which lie on the transition path to another local minimum. Therefore, the barriers between different globular substructures and the coil ensemble are smeared out. To illustrate this matter, we exemplarily show a comparison of the distribution of *R*_g_ in the conformational substates at *T* = 280 K in the supporting information. Indeed, we identify pronounced local minima in the globule region at most temperatures, nonetheless. This leads to the assumption that some local minima correspond to semistable structures. Therefore, the "*folding*" of some globular structures may be a multistep process. In the following sections, we provide a more detailed discussion of the conformational substates. We want to emphasize that the abovementioned smearing-out may only be visible in case of extensive sampling because the conformational diversity may not be captured otherwise. Furthermore, we want to emphasize that the actual kinetics, as we have quantified them with the MSMs, cannot be captured in this projection. Conclusively, *R*_g_ is not an optimal collective variable for the evaluation of the kinetics of the CGT. Consistently, the same has been found for protein folding processes. Indeed, using *R*_g_ as a reaction coordinate to this end may lead to an underestimation of the protein folding barrier as well.^19,21^

The CGT temperature can be obtained straightforwardly from the equilibrium constants. We estimated this property to be T* = 284.2 K, which is consistent with previous computational studies with the same force field.^17,18^ Furthermore, we determined the enthalpy and the entropy of the CGT from the temperature dependence of the equilibrium constant. Thus, we were able to quantify the delicate balance between these two quantities. Clearly, the thermosensitive character of the CGT originates from the increasing impact of the entropy on the free energy at high temperatures. Despite the good agreement with previous computational studies, we report a significant difference of the estimated CGT temperature in comparison to experimental estimations. We expect this deviation to originate from an interplay of inaccuracies in the force field and the water model. In particular, we expect the thermodynamics of solvation to be crucial for the energetic balance (see below). Because the choice of the water model is known to influence the effective force field temperature in simulations,^66^ it is not surprising that a shift in the transition temperature of processes, such as the CGT,^18,67,68^ but also of protein folding^69^ may be observed. Several attempts have been made to optimize a force field to correctly reproduce the experimental LCST behavior of this system.^67,70^ However, we decided to use the OPLS-AA force field, because, in contrast to NIPAAM-specific force fields, it has been validated to qualitatively reproduce the thermosensitive character of a series of different polymers.^18^

Furthermore, we were able to quantify the entropy difference of coil and globule at different temperatures. Counterintuitively, we found that the entropy of coil and globule are of very similar magnitude. From naively looking at simulations of this system—especially with short simulation time—the globule state may potentially be expected to be of significantly lower entropy than the coil state. This deception may be resolved as follows: commonly, we imagine high entropy states to be very dynamic and somewhat *floppy*. Due to the long lifetime of the globule conformations, we would usually not expect this state to be high in entropy. However, this state consists of many of these conformations and the entropy is actually characterized by the *number of microstates that contribute to a certain (macro)state,*(71−74) rather than by the lifetime or dynamics of these microstates. Therefore, it is comprehensible that the entropy of both conformational states—that is, coil and globule—is of similar magnitude.

Generally, we note that Δ*S*_Pol_ · *T* is rather small in comparison to our estimate of the total entropy of the CGT. Conclusively, we expect other contributions to the entropy to be important. This may, for example, include entropic terms of higher order or terms including the solvent. Consequently, we expect the solvation entropy to be of major importance for the energetic balance of the CGT.

We quantified the transition rates of both the CGT and the back transition, that is, the GCT. Generally, these rates are of similar magnitude. However, their temperature dependence differs. We note that the transition rate from globule to coil is generally low at all temperatures and only changes moderately with increasing temperatures. In contrast, the transition rate from coil to globule continuously increases. Accordingly, below the CGT temperature, the transition from coil to globule is rarely sampled. In addition, the rate of the back transition is generally low. Therefore, especially at low temperatures, a substantial amount of simulation time is necessary to estimate the equilibrium constant of the process. We want to emphasize that the kinetics of longer polymer chains may generally be expected to lie on even slower time scales, unfortunately. Therefore, achieving results of comparable accuracy for as many temperatures may be infeasible for longer chains lengths with the current computational resources.

These findings seem to be inconsistent with the projection of the free energy on *R*_g_, which we show in Figure 1. There, hardly any barrier between the two states is visible. However, we explain this apparent inconsistency as follows: Looking merely at *R*_g_, the free energy barriers between the rather broad local minimum of the coil conformations and the different globule minima are being projected onto each other. Yet, we were able to resolve this issue by distinguishing kinetically separable structures with the number of segment contacts and the sum of segment angles (see below).

Generally, we found that accurate estimations of kinetics and thermodynamics indeed require a substantial amount of sampling. After all, the obtained results still show uncertainties and deviations from the expected trend. We believe that these deviations may be explained by the fact that we did not capture the whole conformational diversity at all temperatures (see below). Nevertheless, we want to emphasize that not every quantity requires an equal amount of sampling to be estimated in an equivalent precision. Undoubtedly, the mean *R*_g_ at a certain temperature—which is a useful descriptor for the CGT—may converge in a substantially shorter simulation time.

### Conformational Description at Different Temperatures

Generally, the stiffness of polymer chains to a certain degree depends on the temperature; consequently, equally does its persistence length. Therefore, modeling the polymer as a freely jointed chain with constant segment length over a wide range of temperatures may potentially lead to a small bias. For this reason, we chose to model the polymer with generally comparably short segments. Hence, we used the lower boundary within the range of uncertainty of the estimation of the persistence length in prior publication.^18^ Thus, our model is expected to be optimal for simulations at *high* temperatures. At low temperatures, our model might theoretically overestimate the flexibility of the polymer. However, we do not expect this circumstance to cause a large bias, because—in the worst case—it may lead to generally smaller bending angles. *Underestimating* the flexibility on the other hand may result in the model blurring conformationally relevant bends. Because the obtained conformational states are extremely consistent over the range of invested temperatures, we are confident in the choice of the length of the segments.

Consistent and comprehensive assignment of the conformational substates at different temperatures is rather challenging. Clearly, these substates may generally be modeled with various levels of detail. Indeed, models of different degrees of detail are neither wrong, nor right, because the MSM generally provides kinetically separated states. However, we aimed on building consistent, yet independent models at different temperatures with the smallest necessary number of reference states. Due to the high consistency over this wide range of temperatures, we believe that we succeeded in building models of sufficient detail, without overcomplicating the conformational modeling.

One of the biggest challenges was the modeling of *G0* because we only discovered it at two temperatures, that is, 285 and 295 K. In fact, it is located rather differently at these two temperatures. Possibly, the outlier states at these two temperatures might even be modeled as independent distinct outliers eventually. Interestingly, these two temperatures both consistently show a rather high deviation from the trend in all preceding analyses. Further, the uncertainty of the thermodynamic and kinetic quantities is conclusively high at these two temperatures. Accordingly, we believe that these two anecdotal conformational states, which we did not sample at other temperatures, exhibit a particular influence on the thermodynamic and kinetic evaluation. We hypothesize that the *G0* state is surrounded by a rather high energetic barrier and is therefore *hard to reach.* We want to emphasize here that it is still not highly occupied and that transition *to* this state and *out of* this state have been observed. Therefore, it may *not* be interpreted as a *free energy sink* in terms of a global unescapable minimum.

Ultimately, the freely jointed chain model enabled us to resolve conformational substates. Indeed, we found that resolving substates solely by means of *R*_g_ and σ is not successful. Accordingly, we conclude that ν and Ω are important to resolve kinetically relevant degrees of freedom. Furthermore, we note how badly separated the conformational substates are in terms of *R*_g_. Thus, it becomes clear why *R*_g_ is an insufficient collective variable to describe the CGT in terms of kinetics. Undoubtedly, the closeness of these states leads to the concealment of actual transition barriers in the projection of the free energy on the *R*_g_ (Figure 1). Accordingly, conformations which lie in a local minimum of a specific globular structure are projected onto the same *R*_g_ as other conformations which lie on a transition path (to a *different globule*). In other words, conformations which are *somewhat probable* are projected onto conformations which in contrast are rather improbable. Hence, the transition barrier is largely underestimated in this projection of the free energy. Accordingly, this one-dimensional projection on *R*_g_ is misleading due to the blurring of barriers in kinetically important orthogonal degrees of freedom.

## Conclusions

Having invested a substantial amount of simulation time, we were able to sample the conformational dynamics of the NIPAAM 20-mer at different temperatures with superior accuracy. This exhaustive sampling allowed us to reliably estimate the variability of the conformational ensemble. Furthermore, due to this extensive sampling, we were able to reconstruct thermodynamics and kinetics of the CGT of the polymer at different temperatures. We found that while the back transition, that is, globule to coil, generally underlies slow transition times at all temperatures, the frequency of the CGT significantly increases with temperature. In addition, we were able to quantify the balance between enthalpic and entropic contributions to the free energy of the CGT and determined the entropy to be the key quantity for the energetic balance.

Moreover, we were able to compute the entropy difference of the polymer in the coil and globule ensembles at different temperatures. Counterintuitively, this entropy difference is *not large*. This result demonstrates the main difference between the CGT and protein folding: the conformational diversity and therefore the *number of microstates* is large in *both* states. Thus, the entropy of coil and globule is of similar magnitude. Further, we found that the conformational entropy of the polymer alone cannot explain the whole entropic impact on the free energy. Conclusively, we identified the solvation entropy to be of crucial importance for the CGT.

Modeling the polymer conformations as a freely jointed chain provides the possibility to dramatically decrease the dimensionality of the space to be investigated. With this method, we were able to evaluate the conformational diversity of both states, that is, coil and globule. Moreover, we were able to confirm the existence of conformational substates, which consistently reoccur at different temperatures. Furthermore, this revealed the existence of kinetically separable states, which are indistinguishable by means of the radius of gyration alone, which is often the sole identifier for conformational states in such polymer systems. Conveniently, any linear polymer may be described by means of the freely jointed chain model. Conclusively, this description may potentially be used for enhanced simulation methods to overcome the prevalent sampling issue of the CGT.

## Supporting Information Available

An introductory visualization of the CGT, the number of simulation replicas and the total simulations time at the respective temperatures, the estimated Δ*G* from the distributions of *R*_g_, the estimated conformational entropy of the polymer at different temperatures, the applied lag times for the building of the MSMs, an alternative estimation of enthalpy and entropy at different temperatures and the linearized van’t Hoff plot of the equilibrium constants, the distribution of *R*_g_ in the observed conformational substates at 280 K, the convergence of the estimated Δ*G* for increasing simulation time at different temperatures, and the time series of *R*_g_ from all simulation replicas at all simulations temperatures (PDF)

## Abbreviations and Symbols

NIPAAM: *N*-Isopropylacrylamide
CGT: Coil-Globule transition
LCST: Lower critical solution temperature
MSM: Markov state model
*R*_g_: Radius of gyration
σ: Solvent accessible surface area
ν: Number of contacts between segments
Ω: Sum of angles between segments
