# Supplementary material for: Implementation of the Freely Jointed Chain Model to Assess Kinetics and Thermodynamics of Thermosensitive Coil–Globule Transition by Markov States
Source: J Phys Chem B. 2021 May 4;125(18):4898–909. doi: 10.1021/acs.jpcb.1c01946 (PMC8154620; doi:10.1021/acs.jpcb.1c01946)
Supplement: Supplementary file 1 — jp1c01946_si_001.pdf [file jp1c01946_si_001.pdf]

Supporting Information:

Implementation of the Freely Jointed Chain Model to Assess Kinetics and Thermodynamics of Thermosensitive Coil-Globule Transition by Markov States

Patrick K. Quoika[1], Monica L. Fernández-Quintero[1], Maren Podewitz[1], Florian Hofer[1] and Klaus R. Liedl[1]*

[1] Institute of General, Inorganic and Theoretical Chemistry, and Centre of Molecular Biosciences
University of Innsbruck, A-6020 Innsbruck, Austria

E-mail: Klaus.liedl@uibk.ac.at

Abbreviations and symbols

C	Coil
G	Globule
CGT	Coil-Globule transition
LCST	Lower critical solution temperature
NIPAAM	*N*-Isopropylacrylamide
MSM	Markov state model
R_g	Radius of gyration
σ	Solvent accessible surface area
ν	Number of contacts between segments
Ω	Sum of angles between segments

Table of Figures

Figure S1 Coil-Globule transition of NIPAAM. 4
Figure S2 Number of simulation replicas and total simulation time at respective temperature. 6
Figure S3 Delta Gs from ratio of the probabilities of R_g values of coil and globule conformations. 9
Figure S4 Entropy of polymer. 10
Figure S5 Lag times for the building of the hidden MSMs at different temperatures. 11
Figure S6 Enthalpy and entropy from different fits of the equilibrium constant at different temperatures. 12
Figure S7 Van't Hoff plot. 13
Figure S8 Distribution of radius of gyration (R_g) in conformational substates at 280 K. 14
Figure S9 Convergence of ΔG at different temperatures. 15
Figure S10 Time series of the radius of gyration of all replicas at a simulation temperature of 230 K. 16
Figure S11 Time series of the radius of gyration of all replicas at a simulation temperature of 240 K. 16
Figure S12 Time series of the radius of gyration of all replicas at a simulation temperature of 250 K. 16
Figure S13 Time series of the radius of gyration of all replicas at a simulation temperature of 255 K. 17
Figure S14 Time series of the radius of gyration of all replicas at a simulation temperature of 260 K. 18
Figure S15 Time series of the radius of gyration of all replicas at a simulation temperature of 265 K. 19
Figure S16 Time series of the radius of gyration of all replicas at a simulation temperature of 270 K. 20
Figure S17 Time series of the radius of gyration of all replicas at a simulation temperature of 275 K. 21
Figure S18 Time series of the radius of gyration of all replicas at a simulation temperature of 280 K. 22
Figure S19 Time series of the radius of gyration of all replicas at a simulation temperature of 285 K. 23
Figure S20 Time series of the radius of gyration of all replicas at a simulation temperature of 290 K. 24
Figure S21 Time series of the radius of gyration of all replicas at a simulation temperature of 295 K. 25
Figure S22 Time series of the radius of gyration of all replicas at a simulation temperature of 300 K. 26
Figure S23 Time series of the radius of gyration of all replicas at a simulation temperature of 305 K. 27
Figure S24 Time series of the radius of gyration of all replicas at a simulation temperature of 310 K. 28
Figure S25 Time series of the radius of gyration of all replicas at a simulation temperature of 315 K. 29
Figure S26 Time series of the radius of gyration of all replicas at a simulation temperature of 320 K. 29
Figure S27 Time series of the radius of gyration of all replicas at a simulation temperature of 330 K. 29
Figure S28 Time series of the radius of gyration of all replicas at a simulation temperature of 340 K. 29

SI figures

Introducing the Coil-Globule Transition of NIPAAM Visually

Below, we show a schematic visualization of the Coil-Globule transition of NIPAAM, Figure S1. We show exemplary structures of coil and globule conformations. There, we also depict the chemical formula of the NIPAAM monomer.

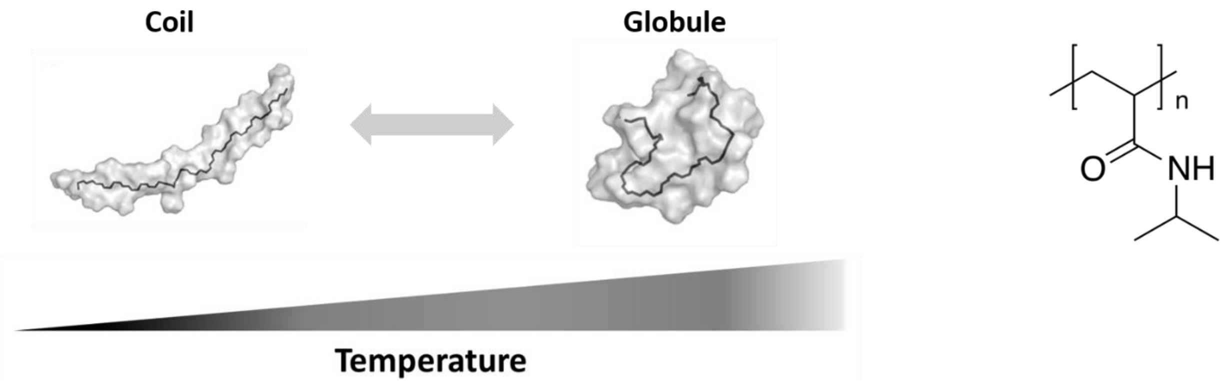

Figure S1 Coil-Globule transition of NIPAAM. On the left we show a sketch of the conformational transition from coil to globule with increasing temperature. Adapted from.[1] On the right hand side, we show the chemical formula of the NIPAAM monomer unit.

Simulation of the 20-mer

While most previous studies focus on the 30-mer of NIPAAM, we chose to simulate the 20-mer for the following reasons: In comparison, the time scale of the transition of the 30-mer is significantly longer. In fact, depending on the temperature, the sampling of the back-transition, i.e., Globule-Coil, underlies waiting times of several hundred nanoseconds for the 30-mer.[1] Therefore, in short simulations it may not be sampled at all. Furthermore, extensive sampling of the conformational dynamics of the 30-mer is even more challenging than for the 20-mer. Generally, the sampling of the conformational space inherently depends on the length of the polymer chain, because the longer a linear polymer chain, clearly, the broader is the conformational space.[2,3] Moreover, the simulation box for simulations of a 30-mer needs to be larger. Consequently, exhaustive sampling of the conformational dynamics is much more expensive for the 30-mer than for the 20-mer. According to previous studies of the dependence of the CGT on the polymer length, the 20-mer is equally suited to draw general conclusions about the process.[4] Since we aimed at obtaining an extensive and diverse conformational ensemble at multiple temperatures, the 20-mer was the natural choice.

Number of Replicas and Total Simulation Time at Different Temperatures

In order to assess the convergence of certain key quantities, we performed longer and more simulations at 280 K. We found that full convergence of the simulations at all temperatures is simply infeasible. Accordingly, we discarded the additional replica and simulation time at 280 K for all analyses.

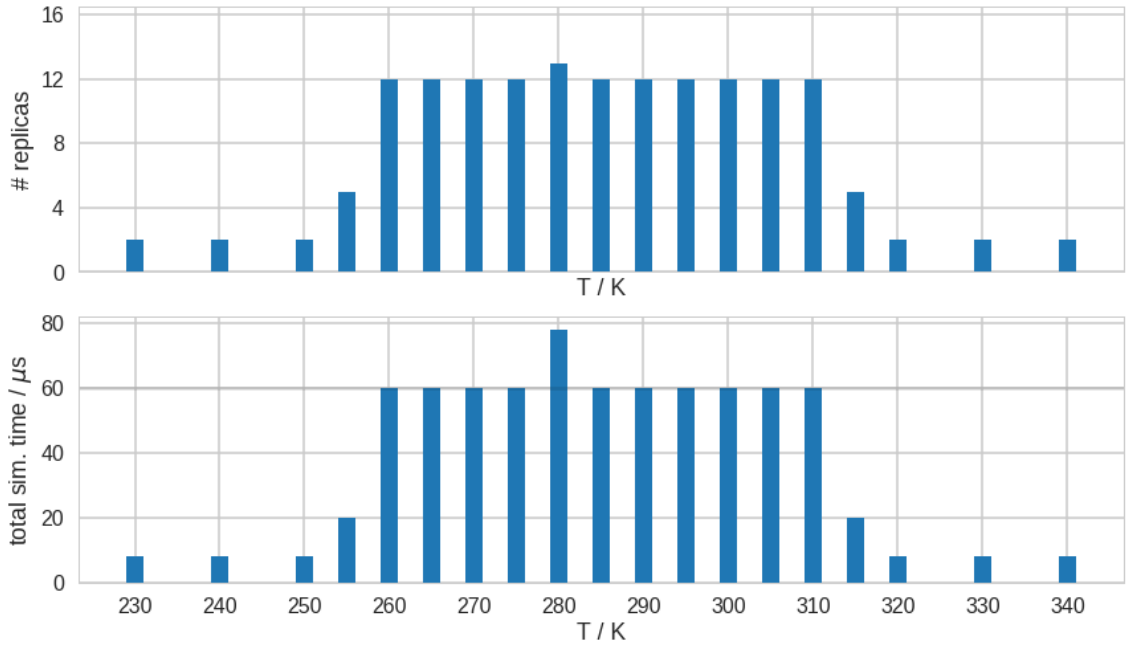

Figure S2 Number of simulation replicas and total simulation time at respective temperature.

State Definition of Coil and Globule

Following a previously published approach, we separated coil and globule conformations in the R_g-σ plane [5]. Accordingly, conformations with low R_g and σ were assigned to be globule, while structures which exhibit high R_g and σ were assigned to be coil. These populations have been separated in the two-dimensional histograms of the conformational ensemble (from all temperatures).

This method has been proven to work robustly for different acrylamide-based polymers, including NIPAAM. However, a small fraction of conformations cannot distinctly be assigned by this method, i.e., conformations with contradicting properties, such as low R_g, but high σ or vice versa. To overcome this issue, we performed hidden MSMs. Therefore, we grouped these *non-distinct* conformations into 250 clusters with the k-means method. Furthermore, we built two-state MSMs from these 252 microstates (including the definite coil and globule structures). As a result, the *non-distinct* conformations were assigned to be either coil or globule and conclusively, we obtained a fully assigned conformational ensemble. We performed this procedure at all temperatures independently.

Segmentation of Polymer Chains

To be able to distinguish between different conformational substates of the polymer chain, we modelled the polymer with the freely jointed chain model. Accordingly, we sectioned the 20mer into 6 segments of a length of 3 monomer units each. Therefore, we did not take the terminal monomer units into account.

We calculated the center of mass of each segment from the positions and masses of all heavy atoms within the respective segment. Furthermore, we calculated distances between these segments. To be able to count contacts between segments, we defined a contact distance between segments in the histograms of segment distances. Therefore, we excluded distances between neighboring segments.

In addition, we modelled the polymer backbone by linear segments. Therefore, we performed a least-square univariate linear spline interpolation. Thus, we fitted the backbone atoms of the segments by linear splines, which are connected at *knots* between segments. As a result, we obtained a continuous representation of the polymer backbone. Therewith, we calculated angles between consecutive segments at the connecting knots. We defined *straight connections* to conform to an angle of zero. Furthermore, we summed up the angles along the polymer backbone. Conclusively, a fully stretched polymer conformation would exhibit small angles between all consecutive segments and therefore a sum of these angles ≈ 0. In contrast, a collapsed polymer conformation would exhibit large angles between consecutive segments and accordingly a large sum of angles.

Free Energies with One-Dimensional State Definition

Here we show the free energy difference of coil and globule at different temperatures from a naïve one-dimensional state definition. Therefore, we separated the conformational ensembles of coil and globule solely in the R_g. We note that these results exhibit a high compliance with the results obtained with the more complex method (see main manuscript). This provides a mutual confirmation of both methods.

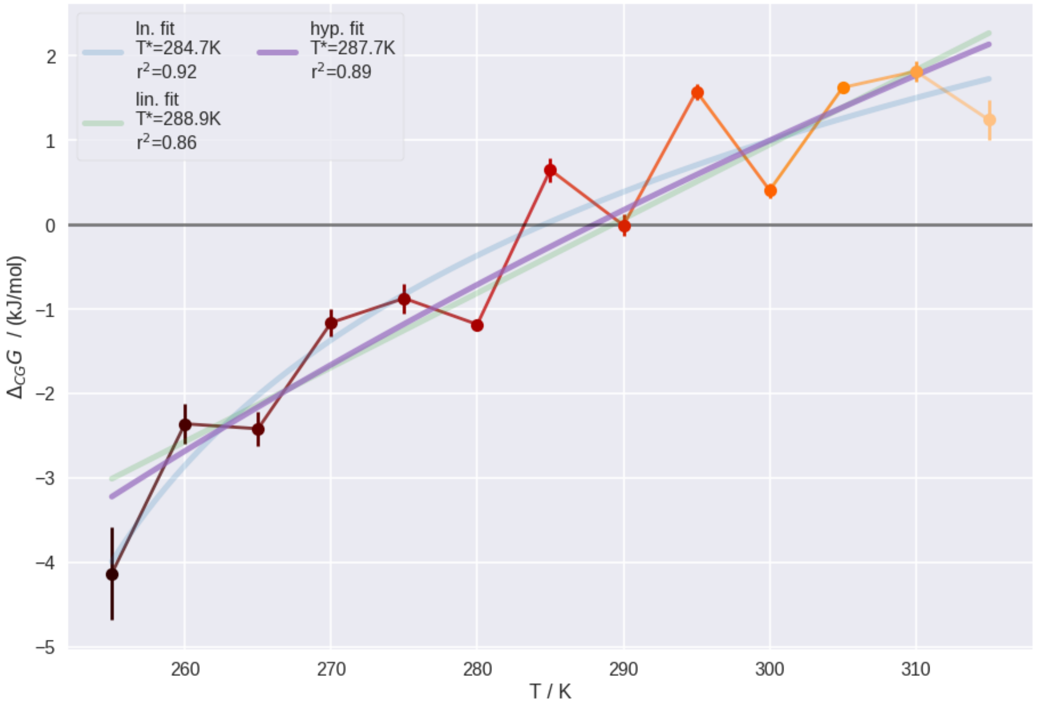

Figure S3 ΔG from ratio of the probabilities of R_g values of coil and globule conformations. Therefore, the ensembles at different temperatures haven been separated solely in the distribution of R_g.

Polymer Entropy within Coil and Globule

We fit the entropies in coil and globule with a hyperbola. Consequently, the respective uncertainties add up (Gaussian error propagation). Furthermore, we subtracted the corresponding interpolated results from each other. Generally, we note that the uncertainty of the entropy for the globule is higher. Furthermore, the higher the temperature the smaller is the deviation from the fit of the coil in comparison to the globule. In addition, we evaluated the trend of the entropic difference of coil and globule at different temperatures multiplied by the temperature.

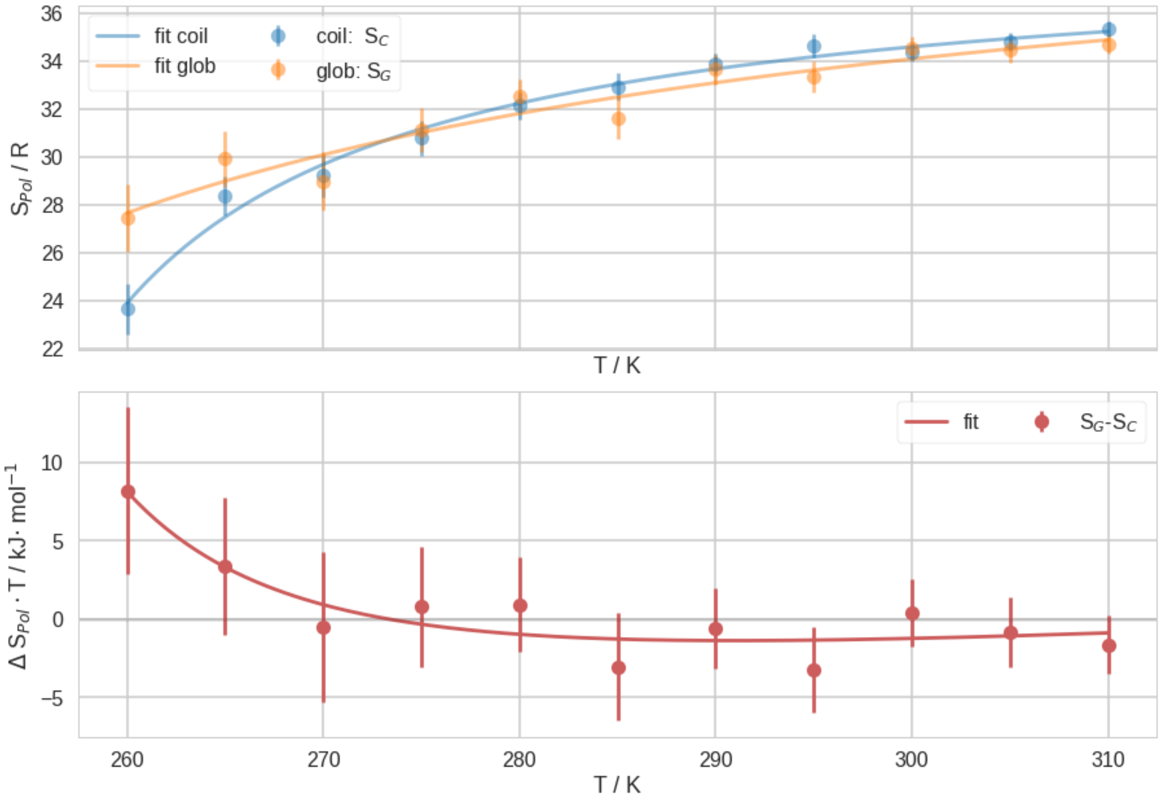

Figure S4 Entropy of polymer. In the upper panel, we show the backbone torsions in the conformational ensembles of coil and globule at different temperatures. We show uncertainties from LOO. In the lower panel, we show the entropy difference between the two conformational states at different temperatures multiplied with the respective temperature, $\Delta S_{Pol} \cdot T$. Uncertainties are calculated as the sum of estimated uncertainties of S_G and S_C.

Lag times for MSMs at Different Temperatures.

Below, we show the lag times we chose for the building of the hidden MSM of the conformational space of the NIPAAM 20-mer. Generally, the higher the temperature, the lower the lag time. This agrees with the expectations of the decrease of the timescales of processes with increasing temperatures, as modelled, e.g., by the Arrhenius equation.

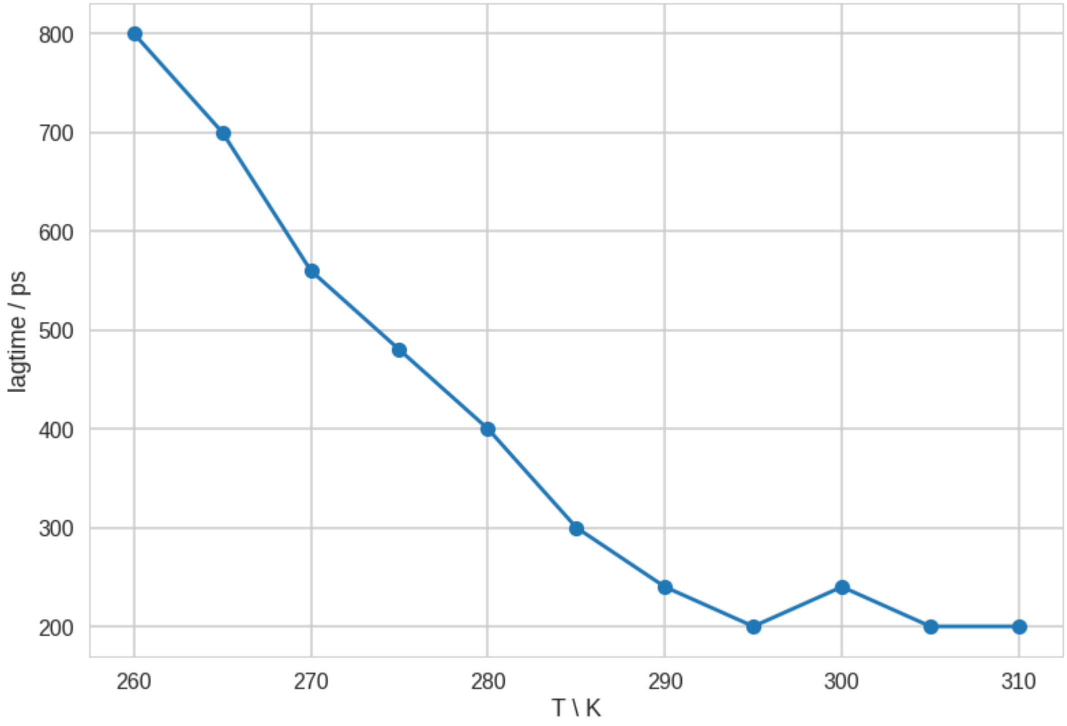

Figure S5 Lag times for the building of the hidden MSMs at different temperatures.

Van’t Hoff Plot and Temperature-Dependent Enthalpy

Generally, it is possible to assume entropy and enthalpy of a process to be constant over a certain range of temperatures. To test this assumption, we also considered the possibility of them being non-constant in an alternative fit. We compare these results in Figure S6. Generally, these fits lead to equivalent results in a certain margin of uncertainty. Furthermore, we linearized the data according to the van’t Hoff equation (van’t Hoff plot), Figure S7. Accordingly, we plot ln(K_{equ}) against the inverse temperature there. Taking a closer look at this linearization, we do not see any significant deviation from a linear trend (no parabolic trend is visible). Conclusively, we assume enthalpy and entropy of the Coil-Globule transition to be approximately independent of the temperature within the range of sampled temperatures in this analysis.

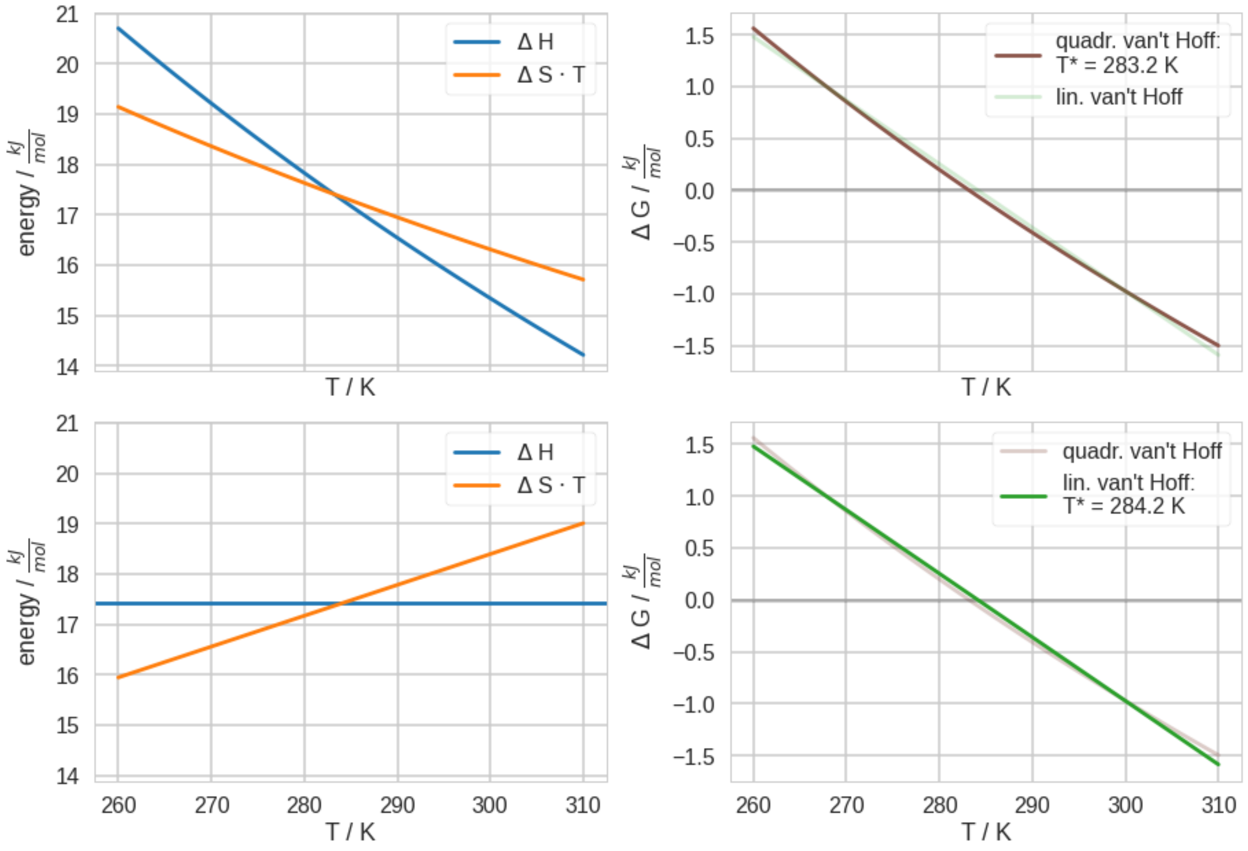

Figure S6 Here we show the resulting enthalpy and entropy from different fits of the equilibrium constant at different temperatures. In the upper panel, we assume both entropy and enthalpy to depend on the temperature. In the lower panel, we assume both to be temperature independent. To facilitate a comparison between the two quantities, we plot the entropy multiplied with the respective temperature.

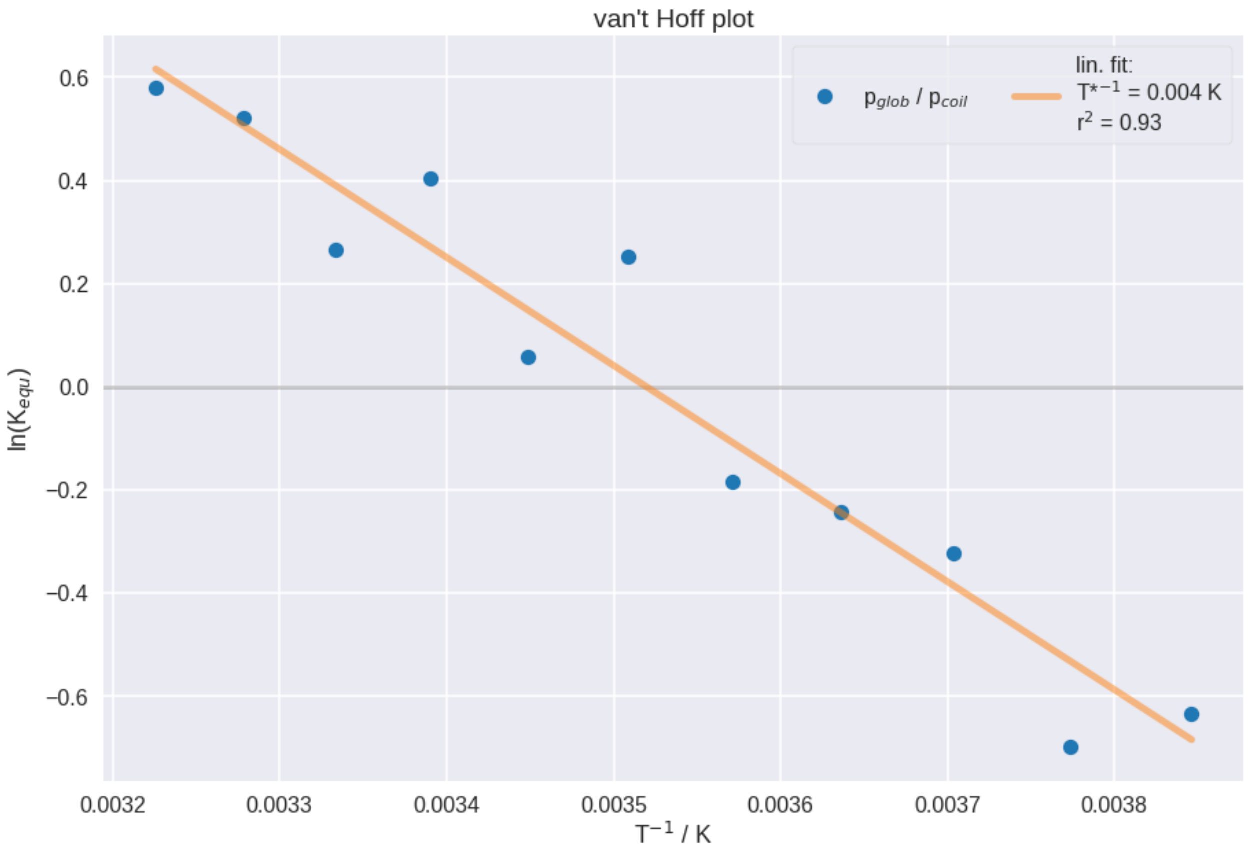

Figure S7 Van't Hoff plot. We ln(K_{equ}) against the inverse temperature. According to the van't Hoff equation we expect to see a linear trend. Conclusively, we assume the enthalpy and entropy to be independent of the temperature within this range of temperatures. In this linear fit, the enthalpy is proportional to the slope of this linear fit, while the entropy is proportional to the intercept.

Radius of Gyration in Different Substates

Below, we show the distribution of R_g in different conformational substates, Figure S8. There, it is apparent, how the distribution of R_g in different globular states is different, but still too close to be easily separated. Indeed to a certain extend they overlap. Accordingly, transition states to one or the other globular state may lie at intermediate R_g between the coil ensemble and this respective state.

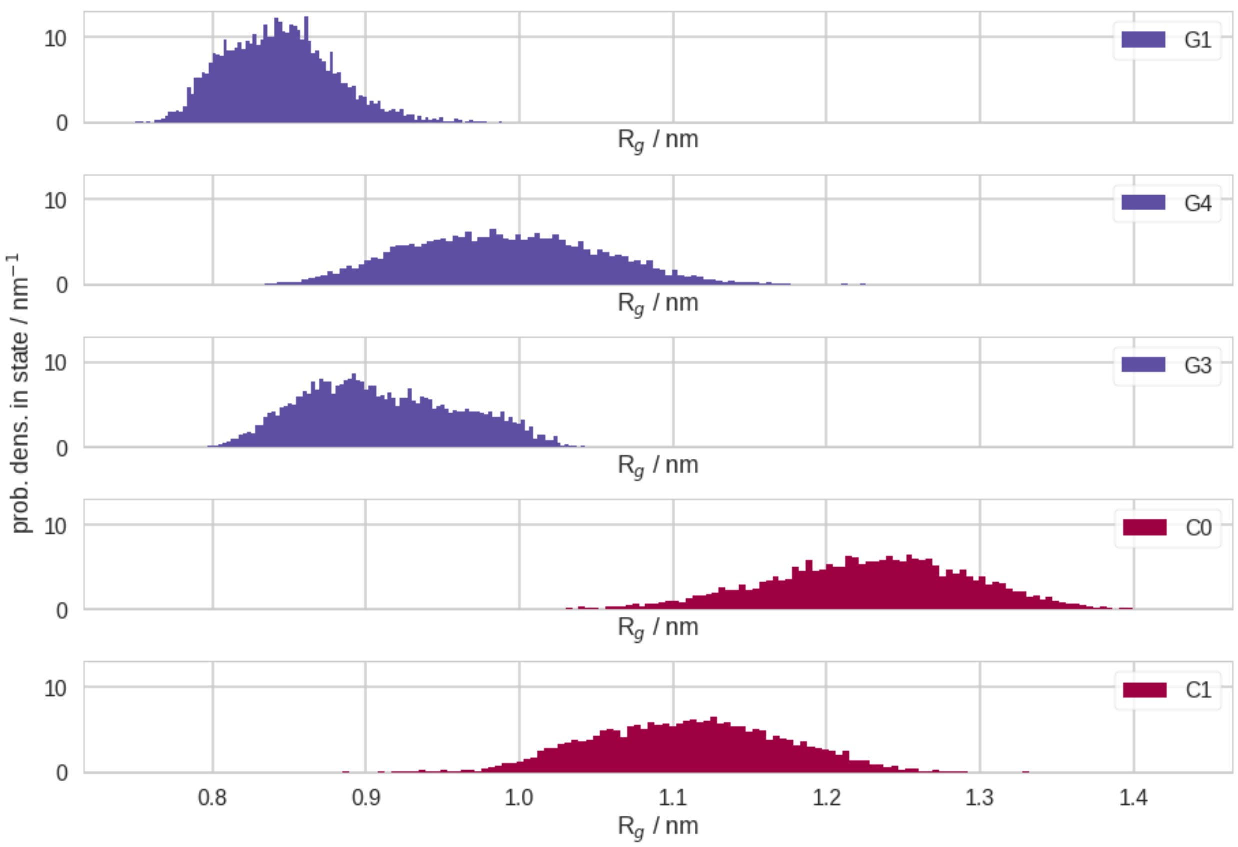

Figure S8 Distribution of radius of gyration (R_g) in conformational substates at 280 K. We show the probability density of R_g in the conformational substates according to the hMSM, which we built from all simulations at 280 K. These models have been built in the space the (R_g, σ, ν, Ω)-space. (See main paper).

Convergence of ΔG at Different Temperatures

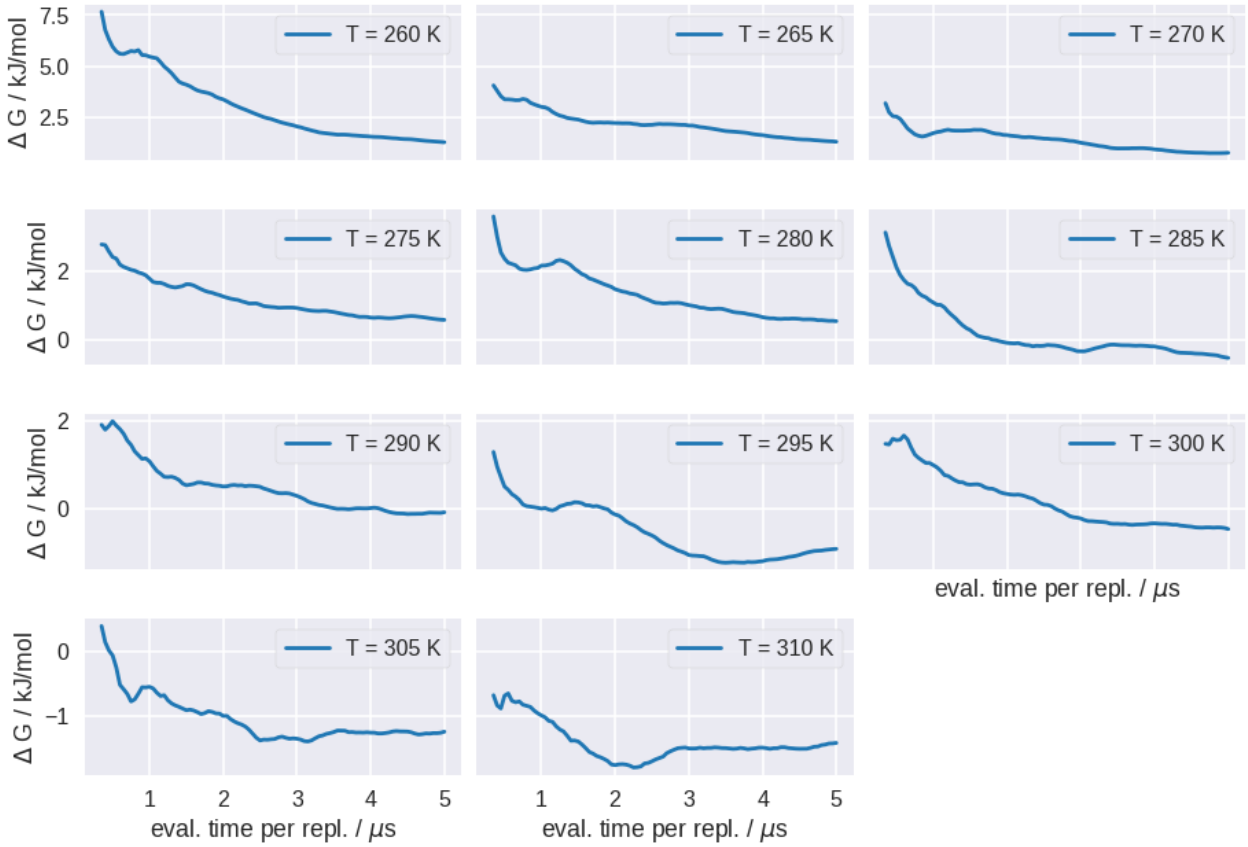

Figure S9 Convergence of ΔG at different temperatures.We show the obtained free energy difference between coil and globule ensemble at different temperatures. To this end, we evaluate increasing simulation per replica at the given temperature and observe the change in ΔG, which we calculate from the obtained equilibrium constant.

Time series of Radius of Gyration

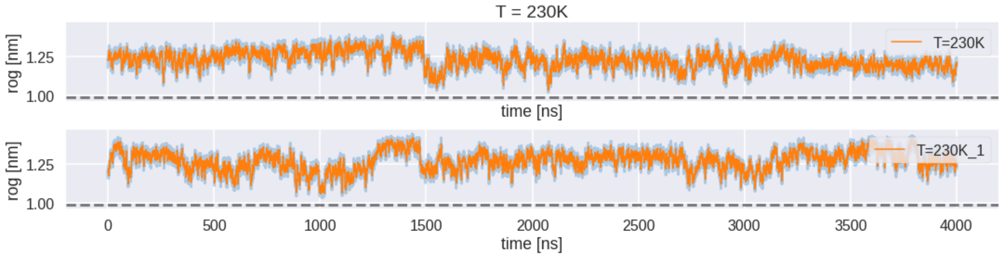

Figure S10 Time series of the radius of gyration of all replicas at a simulation temperature of 230 K.

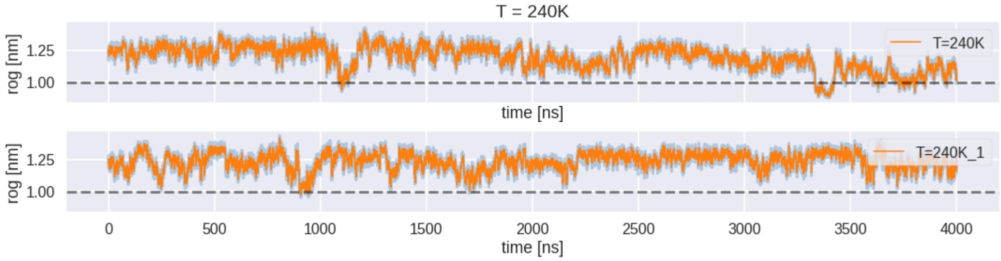

Figure S11 Time series of the radius of gyration of all replicas at a simulation temperature of 240 K.

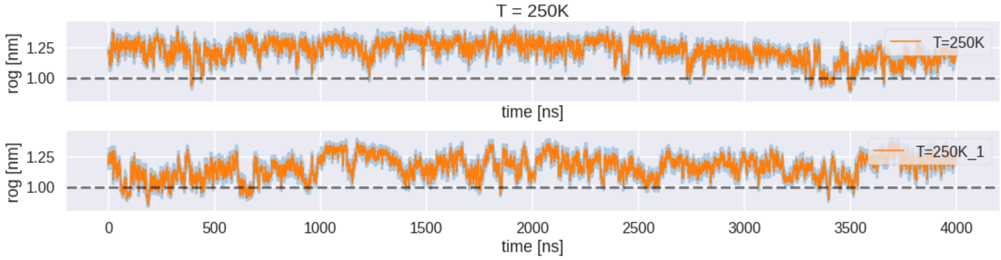

Figure S12 Time series of the radius of gyration of all replicas at a simulation temperature of 250 K.

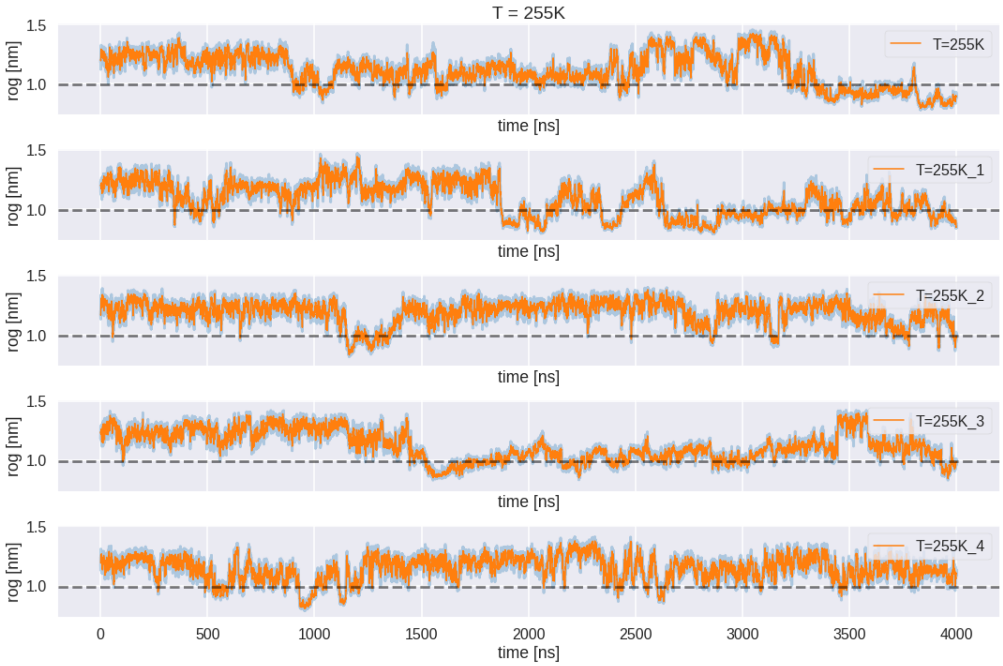

Figure S13 Time series of the radius of gyration of all replicas at a simulation temperature of 255 K.

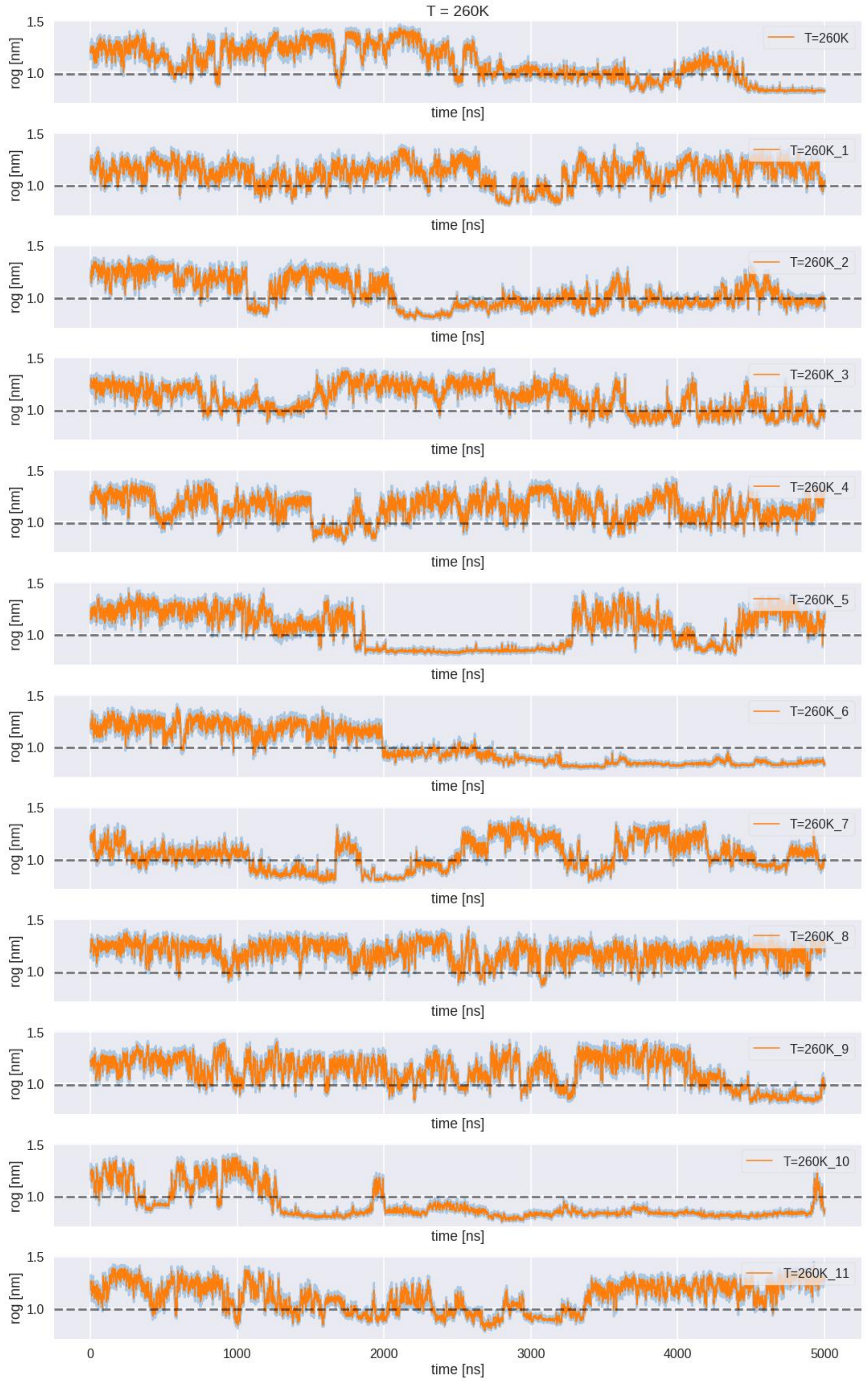

Figure S14 Time series of the radius of gyration of all replicas at a simulation temperature of 260 K.

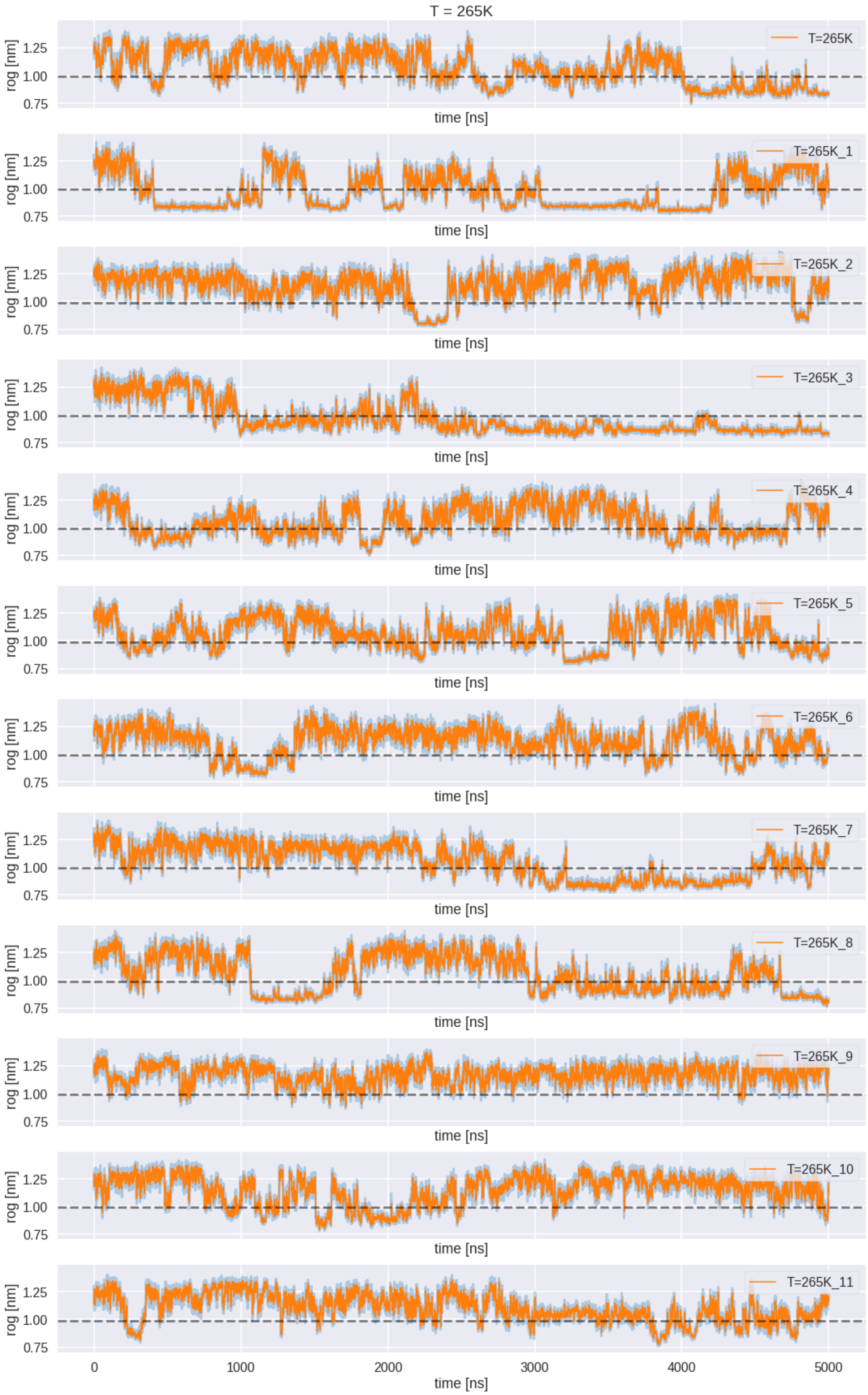

Figure S15 Time series of the radius of gyration of all replicas at a simulation temperature of 265 K.

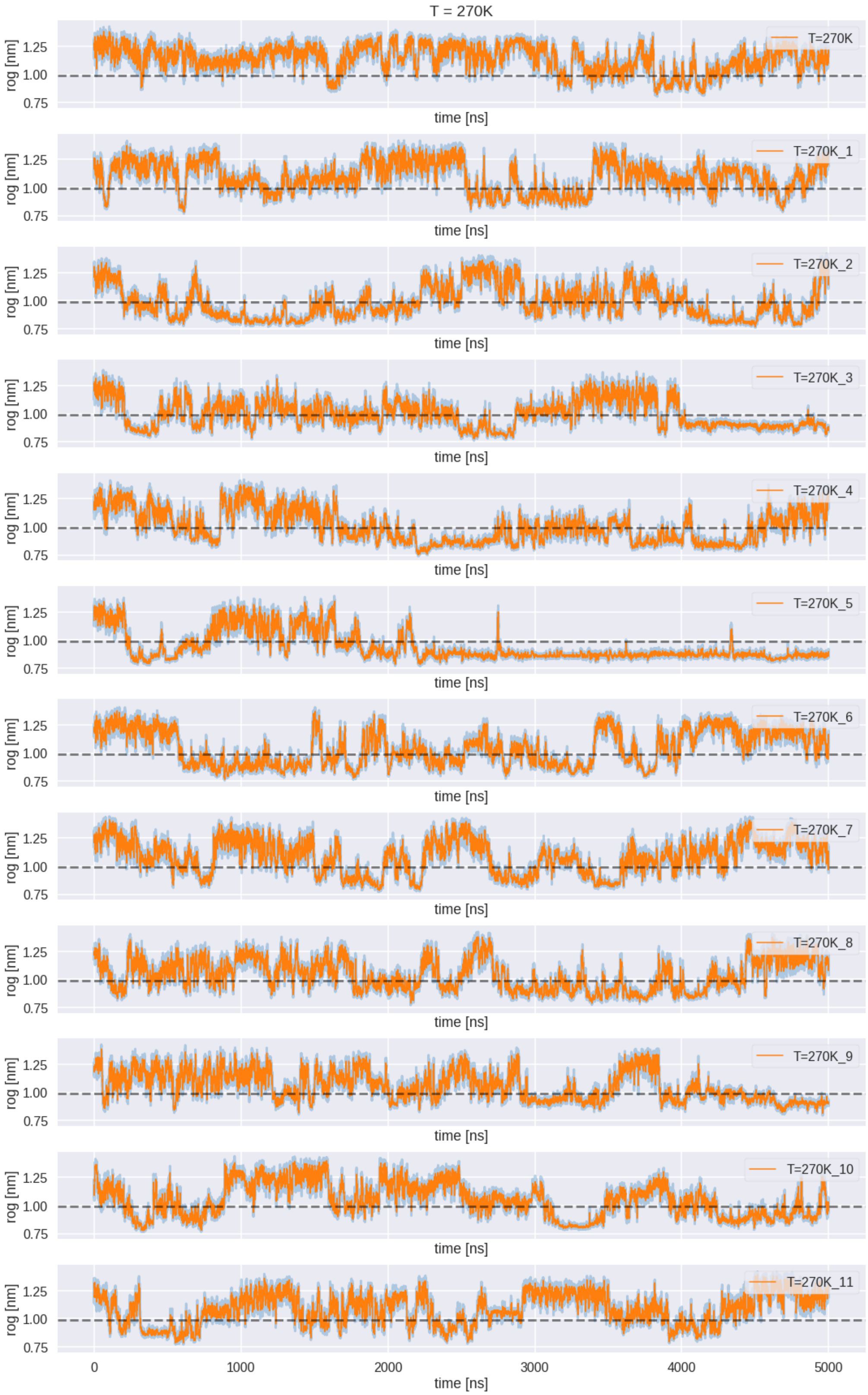

Figure S16 Time series of the radius of gyration of all replicas at a simulation temperature of 270 K.

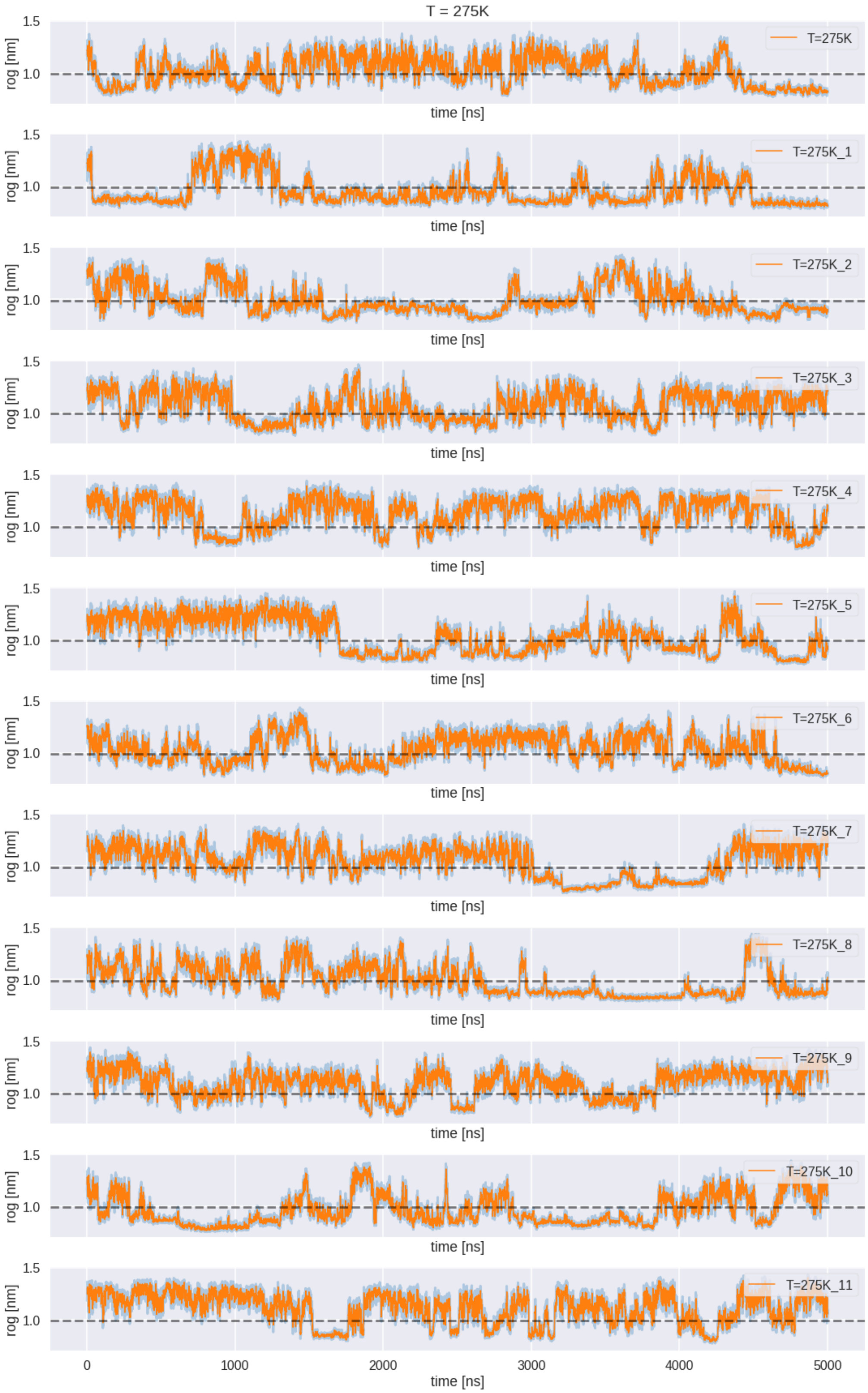

Figure S17 Time series of the radius of gyration of all replicas at a simulation temperature of 275 K.

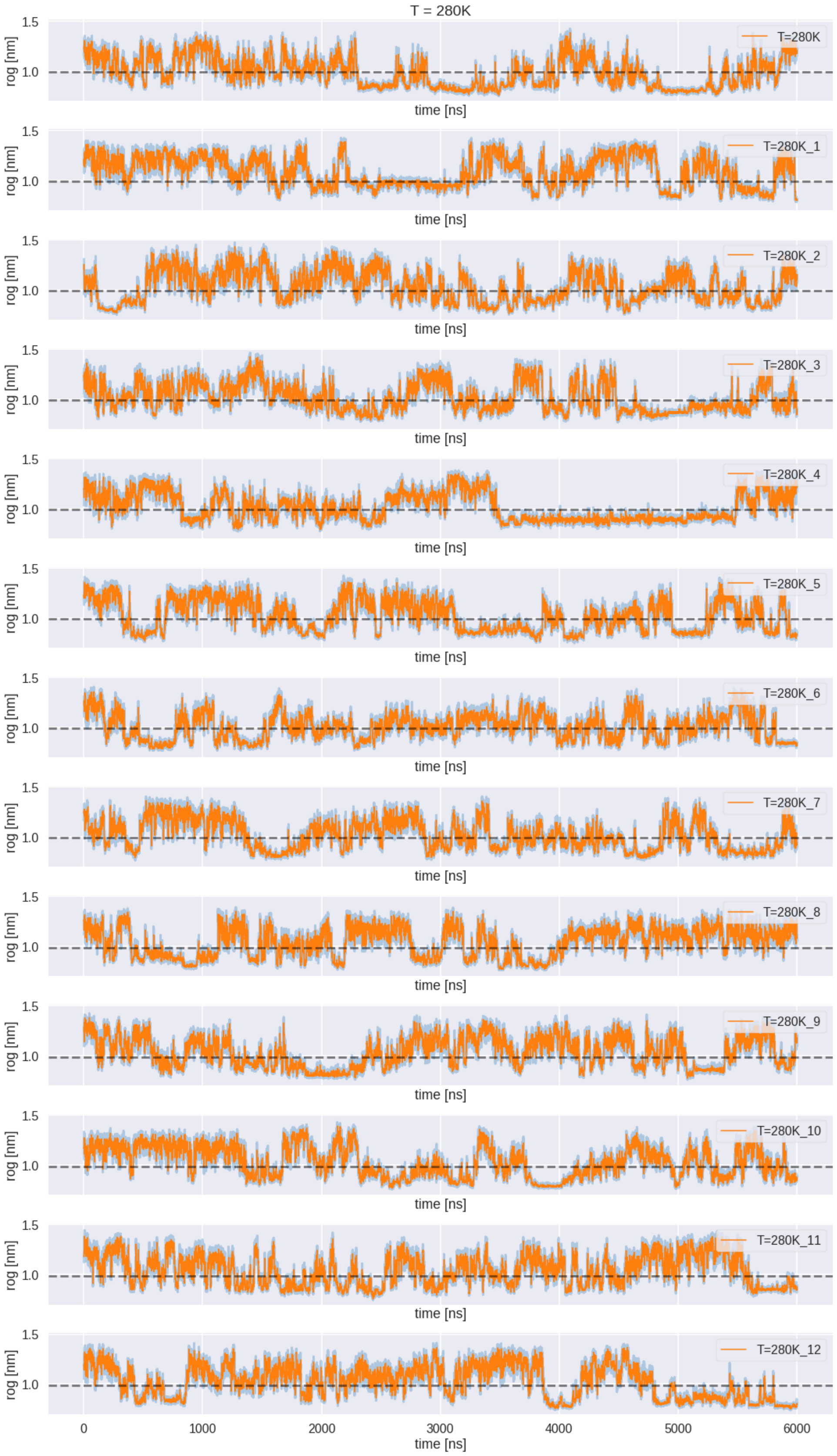

Figure S18 Time series of the radius of gyration of all replicas at a simulation temperature of 280 K.

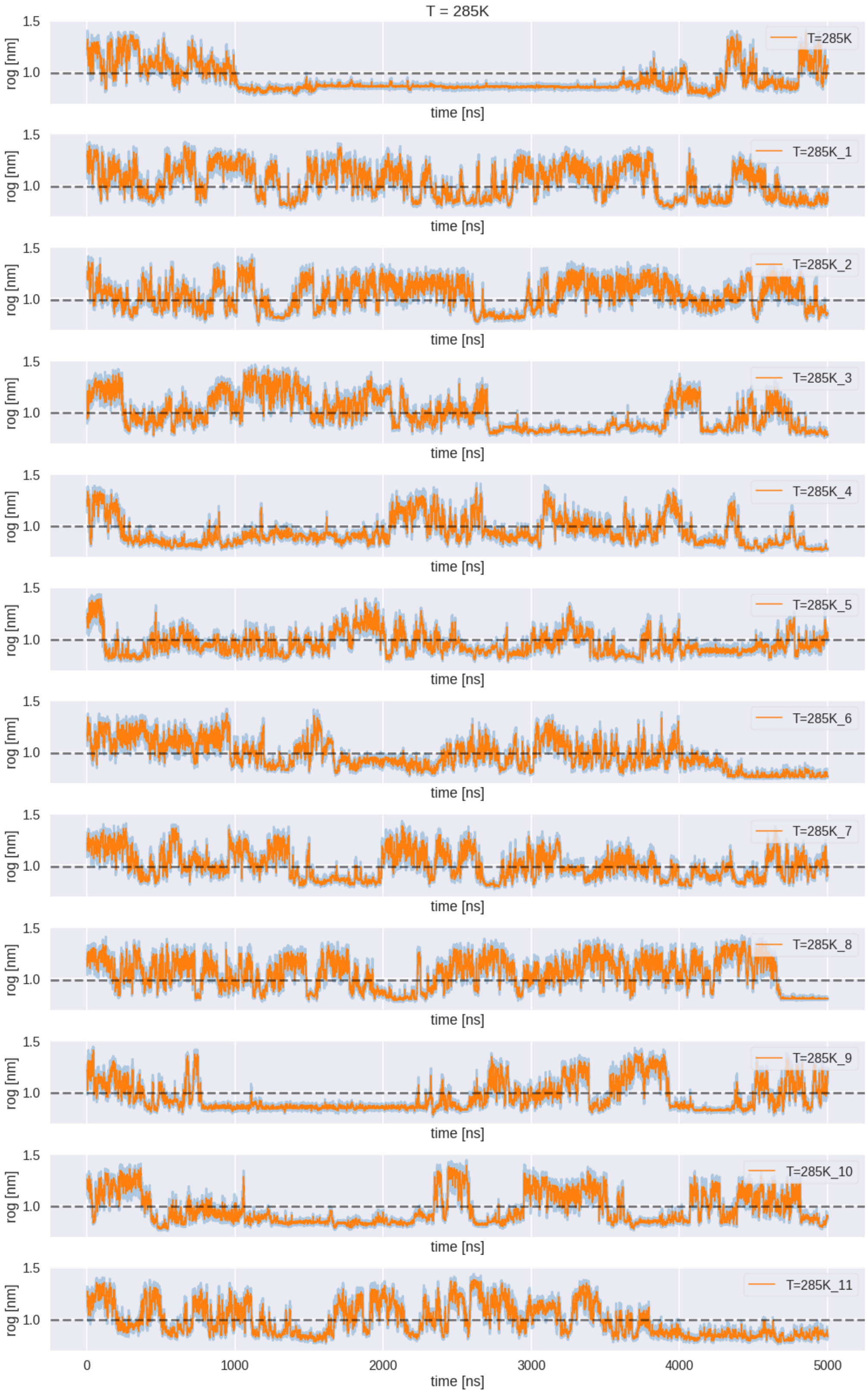

Figure S19 Time series of the radius of gyration of all replicas at a simulation temperature of 285 K.

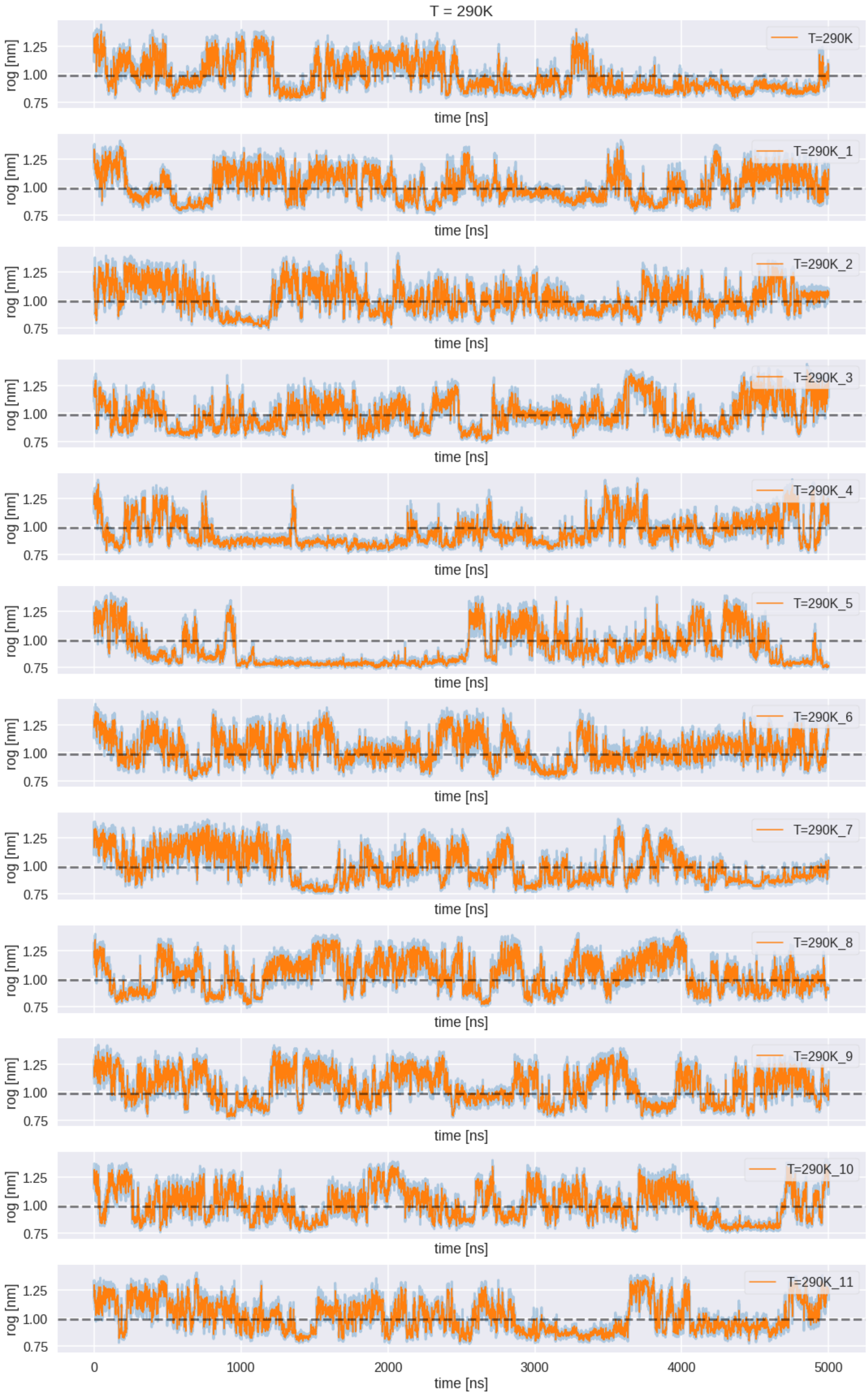

Figure S20 Time series of the radius of gyration of all replicas at a simulation temperature of 290 K.

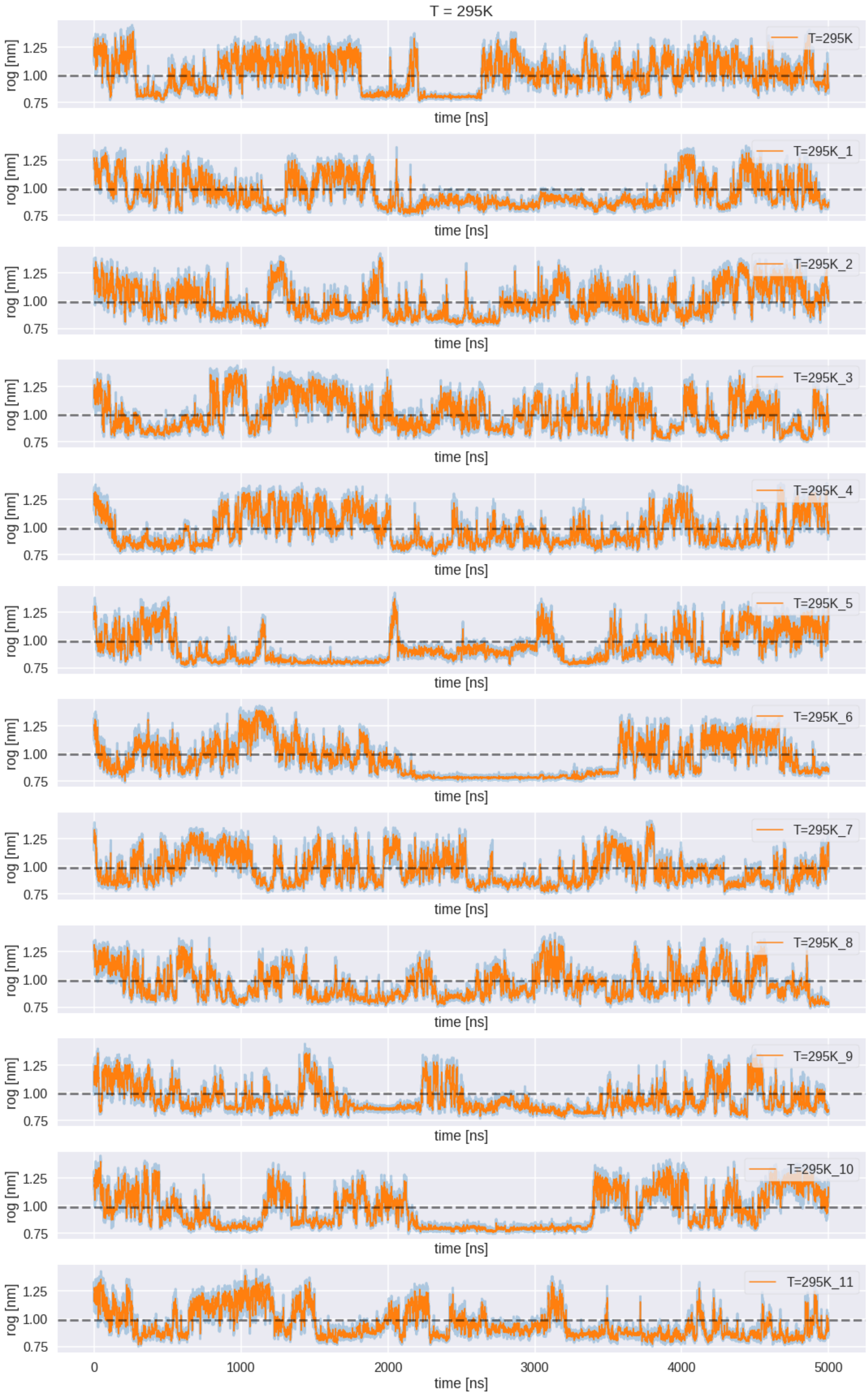

Figure S21 Time series of the radius of gyration of all replicas at a simulation temperature of 295 K.

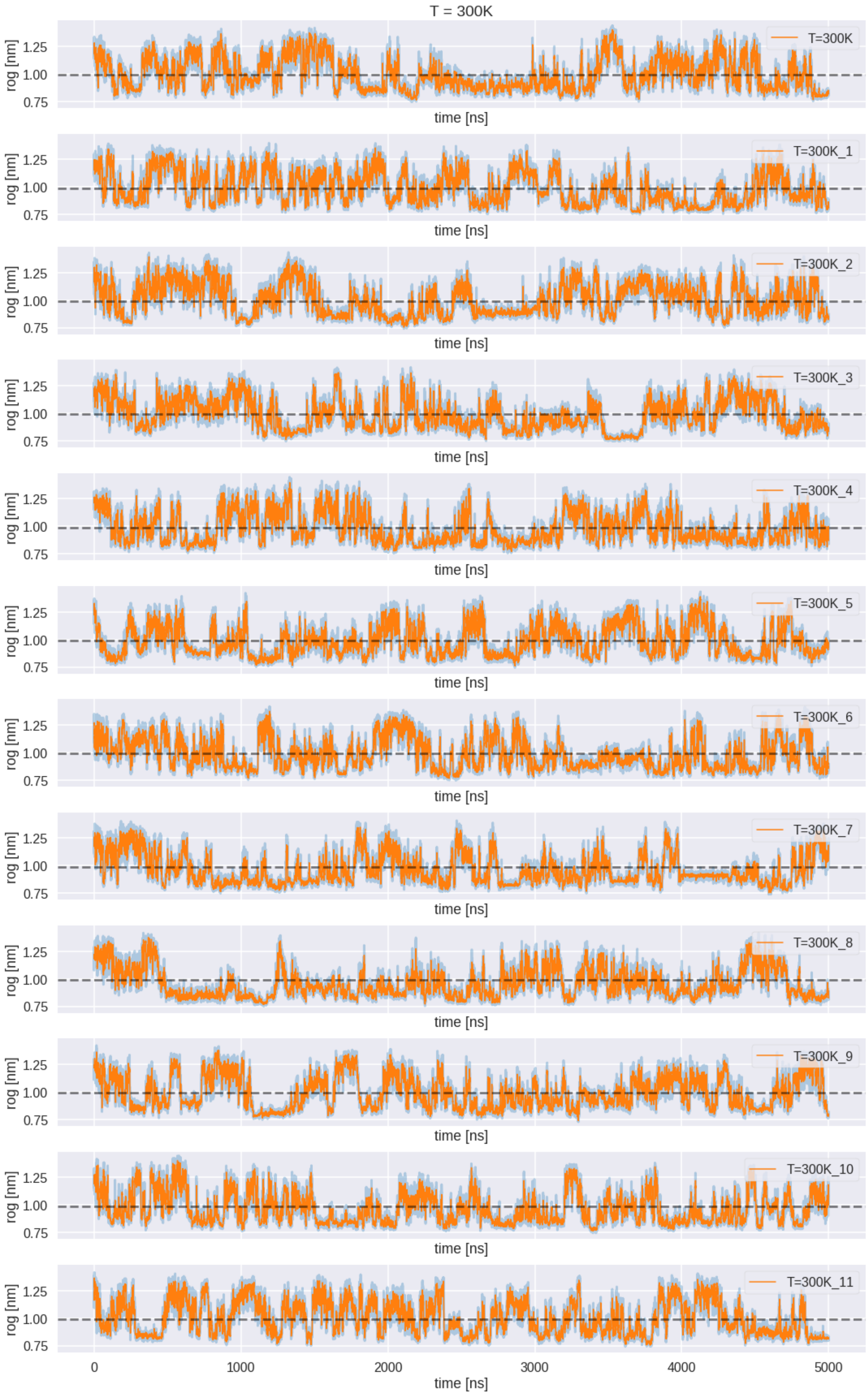

Figure S22 Time series of the radius of gyration of all replicas at a simulation temperature of 300 K.

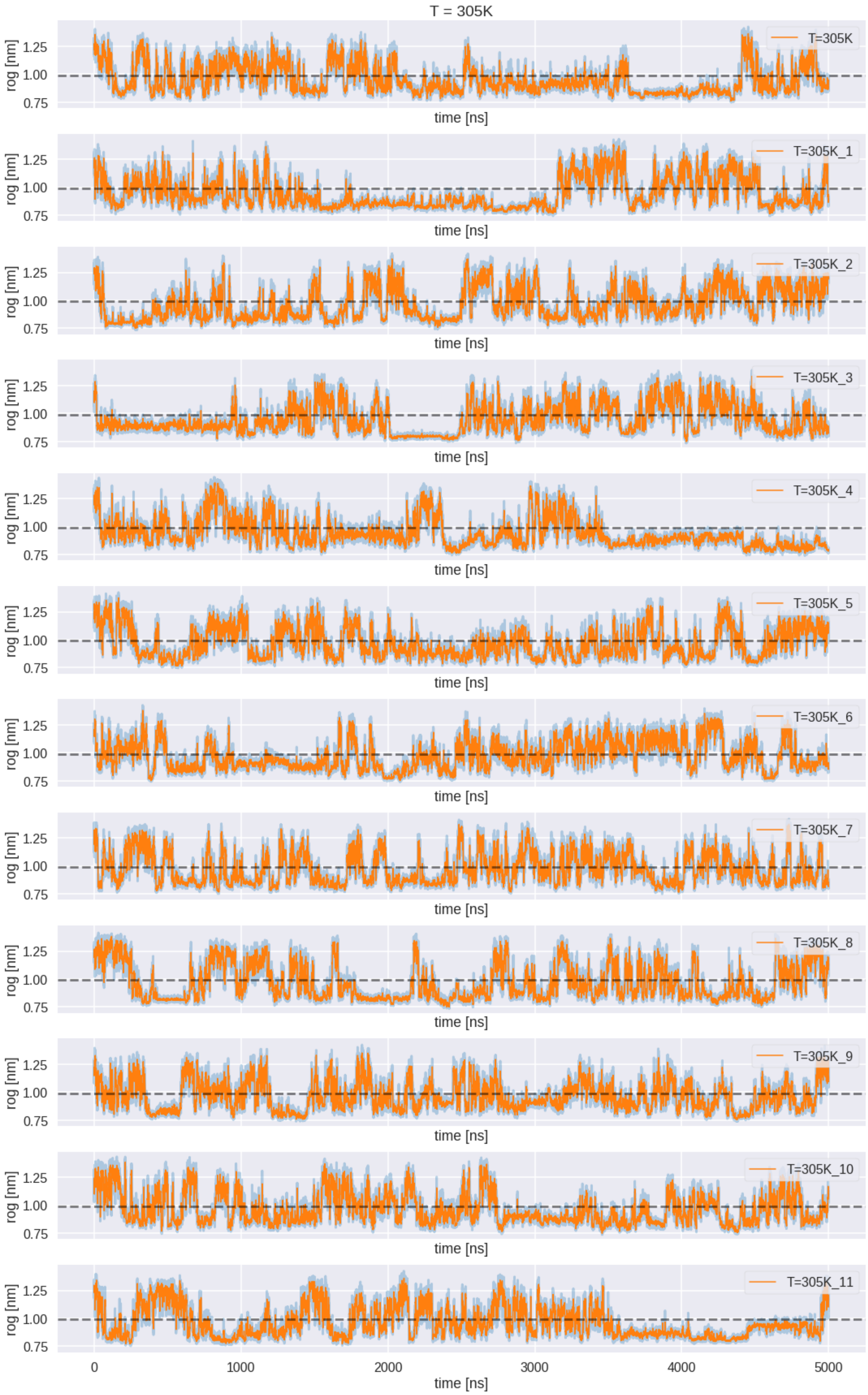

Figure S23 Time series of the radius of gyration of all replicas at a simulation temperature of 305 K.

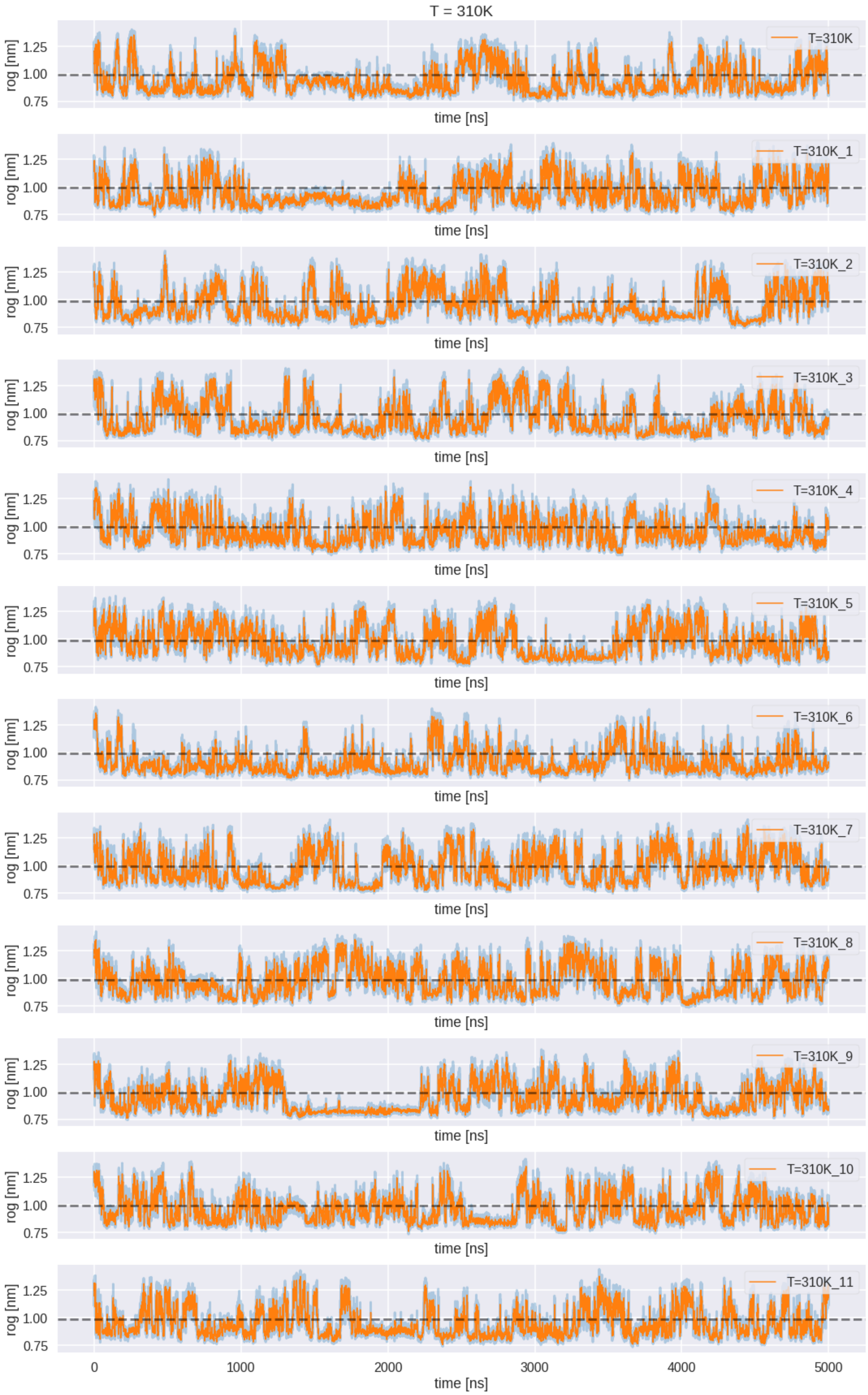

Figure S24 Time series of the radius of gyration of all replicas at a simulation temperature of 310 K.

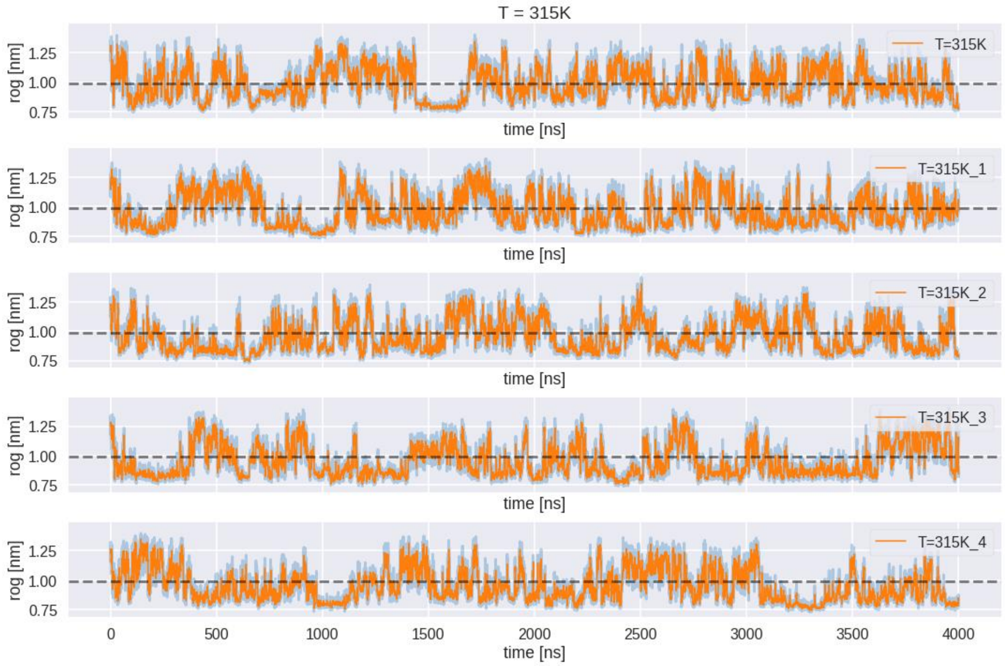

Figure S25 Time series of the radius of gyration of all replicas at a simulation temperature of 315 K.

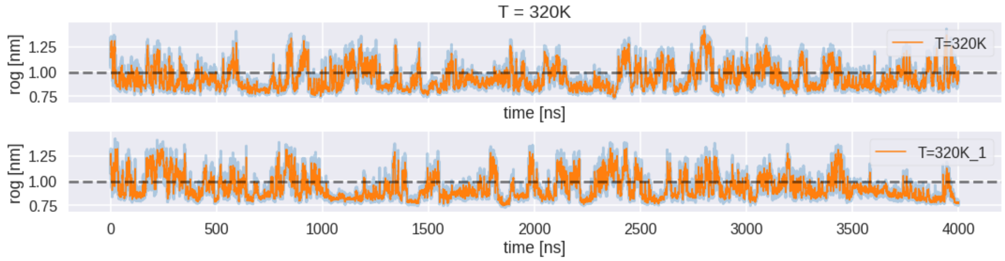

Figure S26 Time series of the radius of gyration of all replicas at a simulation temperature of 320 K.

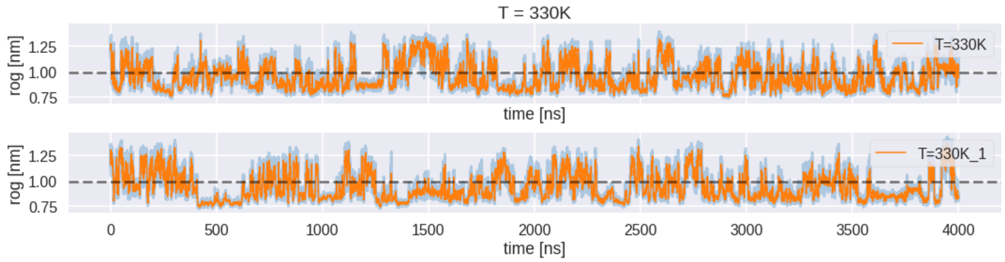

Figure S27 Time series of the radius of gyration of all replicas at a simulation temperature of 330 K.

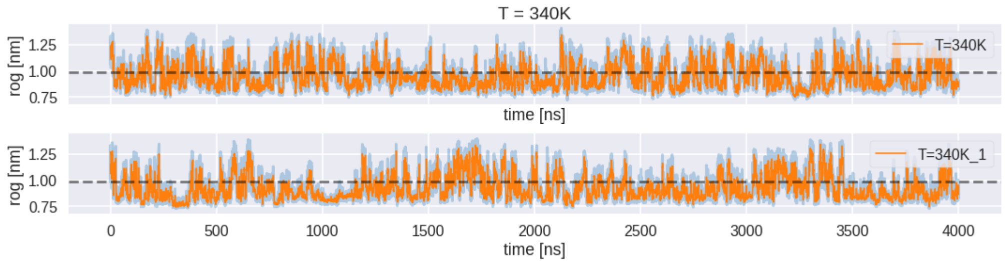

Figure S28 Time series of the radius of gyration of all replicas at a simulation temperature of 340 K.

References

(1) Quoika, P. K.; Podewitz, M.; Wang, Y.; Kamenik, A. S.; Loeffler, J. R.; Liedl, K. R. Thermosensitive Hydration of Four Acrylamide-Based Polymers in Coil and Globule Conformations. *J. Phys. Chem. B* **2020**, *124*, 9745–9756.

(2) Sullivan, D. C.; Kuntz, I. D. Conformation Spaces of Proteins. *Proteins Struct. Funct. Bioinforma.* **2001**, *42*, 495–511.

(3) Levinthal, C. Are There Pathways for Protein Folding? *J. Chim. Phys.* **1968**, *65*, 44–45.

(4) Tucker, A. K.; Stevens, M. J. Study of the Polymer Length Dependence of the Single Chain Transition Temperature in Syndiotactic Poly(N -Isopropylacrylamide) Oligomers in Water. *Macromolecules* **2012**, *45*, 6697–6703.

(5) Podewitz, M.; Wang, Y.; Quoika, P. K.; Loeffler, J. R.; Schauperl, M.; Liedl, K. R. Coil–Globule Transition Thermodynamics of Poly(N -Isopropylacrylamide). *J. Phys. Chem. B* **2019**, *123*, 8838–8847.
